# Reciprocal interactions between malignant cells and macrophages enhance cancer stemness and M2 polarization in HBV-associated hepatocellular carcinoma

**DOI:** 10.7150/thno.87962

**Published:** 2024-01-01

**Authors:** Qingyang Zhang, Yu-Man Tsui, Vanilla Xin Zhang, Anna Jingyi Lu, Joyce Man-Fong Lee, Eva Lee, Gary Cheuk-Hang Cheung, Po-Man Li, Elaine Tin-Yan Cheung, Nam-Hung Chia, Irene Lai-Oi Lo, Albert Chi-Yan Chan, Tan-To Cheung, Irene Oi-Lin Ng, Daniel Wai-Hung Ho

**Affiliations:** 1 Department of Pathology, The University of Hong Kong, Hong Kong.; 2 State Key Laboratory of Liver Research, The University of Hong Kong, Hong Kong.; 3 Department of Pathology, Queen Elizabeth Hospital, Hong Kong.; 4 Department of Surgery, Queen Elizabeth Hospital, Hong Kong.; 5 Department of Surgery, The University of Hong Kong, Hong Kong.

**Keywords:** Hepatocellular carcinoma, Single-cell RNA sequencing, Cancer stem cell, Tumor heterogeneity, Macrophage polarization

## Abstract

**Background:** The tumor microenvironment of cancers has emerged as a crucial component in regulating cancer stemness and plays a pivotal role in cell-cell communication. However, the specific mechanisms underlying these phenomena remain poorly understood.

**Methods:** We performed the single-cell RNA sequencing (scRNA-seq) on nine HBV-associated hepatocellular carcinoma (HCC) patients. The heterogeneity of the malignant cells in pathway functions, transcription factors (TFs) regulation, overall survival, stemness, as well as ligand-receptor-based intercellular communication with macrophages were characterized. The aggressive and stemness feature for the target tumor subclone was validated by the conduction of *in vitro* assays including sphere formation, proliferation, Annexin V apoptosis, flow cytometry, siRNA library screening assays, and multiple *in vivo* preclinical mouse models including mouse hepatoma cell and human HCC cell xenograft models with subcutaneous or orthotopic injection.

**Results:** Our analysis yielded a comprehensive atlas of 31,664 cells, revealing a diverse array of malignant cell subpopulations. Notably, we identified a stemness-related subclone of HCC cells with concurrent upregulation of CD24, CD47, and ICAM1 expression that correlated with poorer overall survival. Functional characterization both *in vitro* and *in vivo* validated S100A11 as one of the top downstream mediators for tumor initiation and stemness maintenance of this subclone. Further investigation of cell-cell communication within the tumor microenvironment revealed a propensity for bi-directional crosstalk between this stemness-related subclone and tumor-associated macrophages (TAMs). Co-culture study showed that this interaction resulted in the maintenance of the expression of cancer stem cell markers and driving M2-like TAM polarization towards a pro-tumorigenic niche. We also consolidated an inverse relationship between the proportions of TAMs and tumor-infiltrating T cells.

**Conclusions:** Our study highlighted the critical role of stemness-related cancer cell populations in driving an immunosuppressive tumor microenvironment and identified the S100A11 gene as a key mediator for stemness maintenance in HCC. Moreover, our study provides support that the maintenance of cancer stemness is more attributed to M2 polarization than the recruitment of the TAMs.

## Introduction

Hepatocellular carcinoma (HCC) is the fifth most common cancer and the third leading cause of cancer-related death worldwide 1. HCCs are often diagnosed at an advanced stage, and tumor recurrence is frequent even after surgical resection 2. In recent years, the emerging combination therapy of tyrosine kinase inhibitors (TKI) and immune checkpoint inhibitors (ICI) has shown some clinical efficacy in the treatment of advanced HCC 3. However, despite the improvements in therapy, treatment of advanced HCC remains a great challenge.

Emerging evidence has indicated that cancer stem cells (CSCs), a subpopulation of tumor cells, bear tremendous ability in cancer initiation, tumorigenesis, metastasis, recurrence and therapeutic resistance 4, 5. The characterization of liver CSCs largely relies on the putative surface markers, such as CD133, CD44, EpCAM, CD90 and CD13 5. Similar to normal organs, tumors also harbor a relatively hierarchical collection of cancer cells and stromal cells, with CSCs residing at the top of the hierarchy 5.

Maintaining an ideal microenvironment or niche is very important for CSCs to protect themselves against stress signals, chemotherapeutic agents, and attacks by the immune system 6. As an indispensable component of the CSC niche, tumor associated macrophages (TAMs) receive immune-suppressive signals from CSCs to further enhance the tumor-promoting microenvironment. Although some studies have already hinted at the crosstalk between CSCs and TAMs 6-9, they have largely relied on traditional bulk-cell strategies or cultured cell lines. Therefore, the precise mechanisms of how the CSCs and TAMs interact remain inadequately understood.

On the other hand, prior studies 10 exploring HCC at the single-cell level have primarily focused on the infiltrating immune cell populations or the intricate HCC ecosystem 11-13. There is still an open question regarding the interaction between CSCs and TAMs in HCC. Herein, we performed single-cell RNA-sequencing (scRNA-seq) in patients' resected HBV-associated HCC samples and identified a previously unrecognized population of CSCs among the cancer cells that exhibited stemness gene signatures and aggressive tumor properties. Taking advantage of the cell-cell communication analysis, we unraveled a strong link between stemness maintenance and macrophage polarization, highlighting M2-like macrophages as a significant contributor to the pathogenesis of HBV-associated HCC. Our results may offer a mechanistic explanation for the variation in treatment responses and highlight the importance of precision medicine.

## Results

### ScRNA-seq profiling of HBV-associated HCC ecosystem and cellular stratification

**Figure 1:**
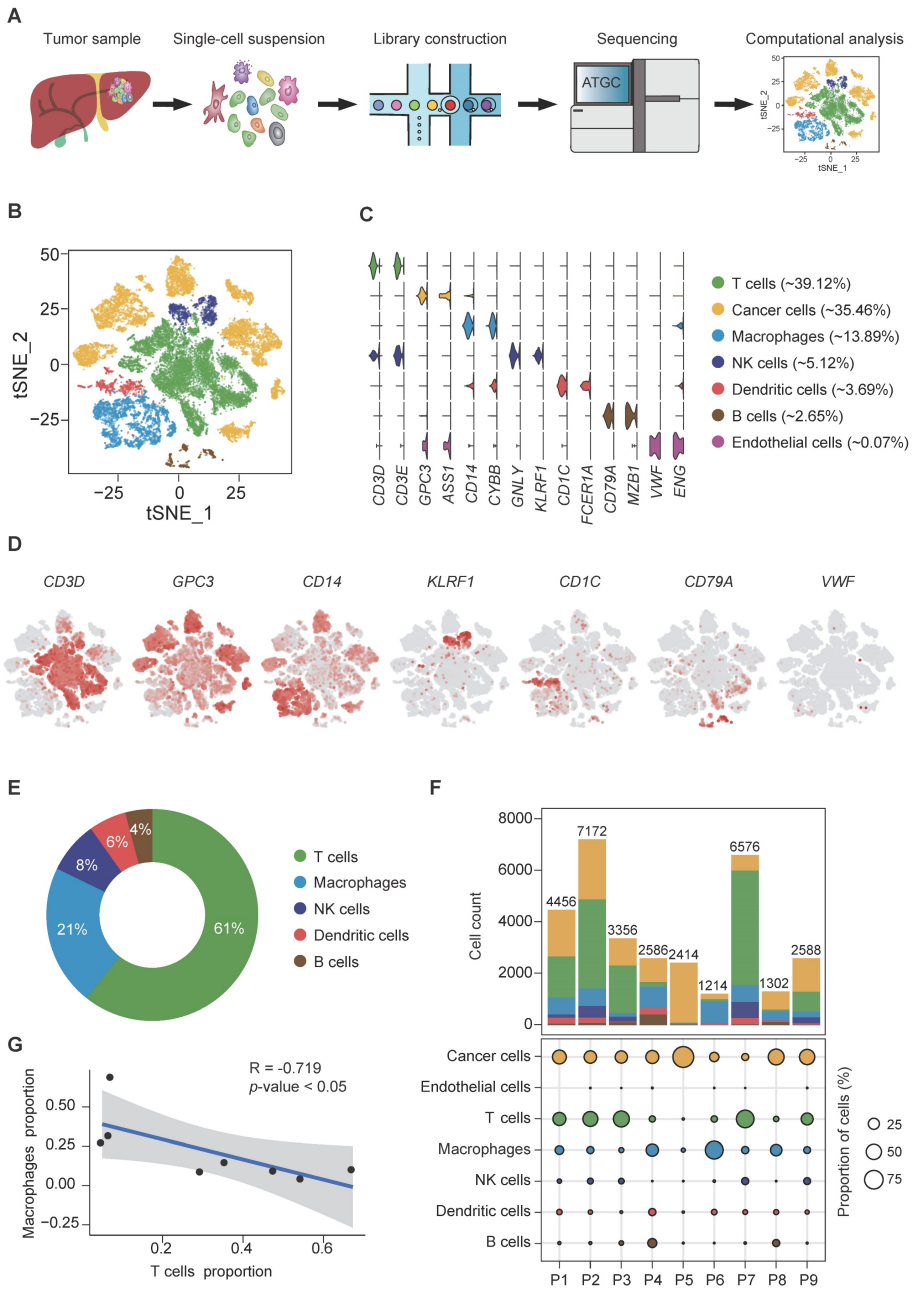
** scRNA-seq of hepatocellular carcinoma revealed major cell types. (A)** Schematic overview of the generation of scRNA-seq data. Nine HCC samples were collected.** (B)** t-SNE plot of 31,664 high-quality cells. Each point is a single cell colored by cluster assignment.** (C)** Violin plots of canonical marker genes expression by cell type with highest log-normalized expression value labeled.** (D)** t-SNE plot, color-coded for expression (gray to red) of marker genes for the major cell types as indicated.** (E)** Representative donut plots depicting distribution of overall immune cell composition.** (F)** The tumor cell lineage compositions inferred by scRNA-seq data of nine HCC sample. The low panel shows the HCC-defined cell types (rows) by patient (columns). The size of the circle represents, for each specific cell type, the fraction of corresponding cell (among the total quality-control passed cells) in each individual. The circles are color coded by defined cell types. The histogram on the top shows, for each individual sample, the accumulation of the raw number of each cell type.** (G)** Linear regression showing the inverse correlation between T cells and macrophages proportion. Pearson's correlation R values are shown and the significance of the difference between two data sets was measured by two-tailed Student's t-test. Only cases with at least 500 non-malignant cells were included.

Fresh, resected HBV-associated HCC tissues from 9 patients were obtained immediately from the operation theaters. All patients were treatment-naïve without chemotherapy or radiotherapy prior to surgery. The demographic and clinical characteristics of the patients are described in supplementary Table S1. Tumors were digested into single-cell suspension, and scRNA-seq was performed using barcoding with unique molecular identifiers (UMIs) (Figure 1A). After the removal of potential doublets and low-quality cells, more than 204 million unique transcripts were obtained from 31,664 cells with more than 29,273 genes captured. To profile the transcriptomic landscape of HCC, unsupervised clustering and t-distributed stochastic neighbor embedding (tSNE) algorithm were carried out. All quantified cells were initially stratified into 44 cell clusters and further assigned to 7 major cell lineages based on the canonical markers and differentially expressed genes (DEGs) (Figure 1B-D, S1A-B). Of the total cells, immune cells (CD45^+^) accounted for approximately 65% (Figure S1C) and were classified into 5 subsets. The most abundant subset was T cells (12,386 cells, marked with CD3D and CD3E), comprising 39.12% of the total cells and over 50% of the total immune cells (Figure 1C, E). Other populations included natural killer (NK) cells (1,622 cells, 5.12% of the total cells, marked with GNLY and KLRF1), B cells (839 cells, 2.65% of the total cells, marked with CD79A and MZB1), TAMs (4,400 cells, 13.89% of the total cells, marked with CD14 and CYBB) and dendritic cells (DCs) (1,168 cells, 3.69% of the total cells, marked with CD1C and FCER1A). Among the non-immune cells, apart from a small population of tumor-associated endothelial cells (21 cells, 0.07% of the total cells, marked with VWF and ENG), the rest were cancer cells (11,228 cells, 35.46% of the total cells, marked with GPC3 and ASS1). Consistent with previous studies 14, immune cells displayed no significant differences among patients (Figure S1C-D, F) at varying proportions (Figure 1F, S1E-F), while cancer cells demonstrated intertumoral heterogeneity and separated according to patients, as expected. Notably, consistent with our previous study 15, we independently observed an inverse correlation in the cell proportion between T cells and total TAMs (Pearson's r = -0.719, *p*-value<0.05) (Figure 1G), implying that TAMs might suppress the tumor infiltration of T cells in HCC.

### Transcriptional heterogeneity of cancer cell clusters and prognostic implications

**Figure 2:**
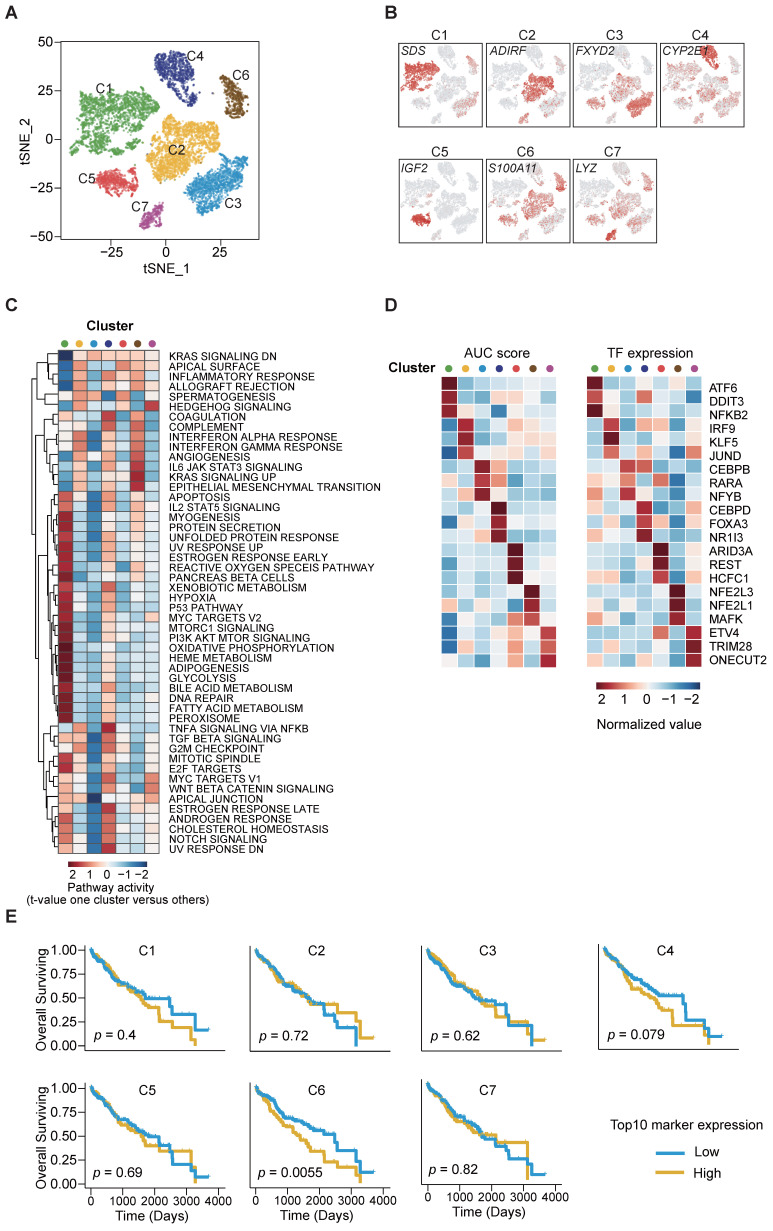
** Identification of cancer cell subpopulations. (A)** t-SNE plot of cancer cells, color-coded by the subsets.** (B)** t-SNE plot, color-coded for expression (gray to red) of marker genes for the subsets as indicated.** (C)** Differential activity pathways in cancer cell subsets (scored by GSVA for each cell). Red represents upregulated pathways; blue represents down-regulated pathways. **(D)** Heatmap showing the regulon activity of cancer cell subsets estimated by SCENIC, depicting the top three transcription factors estimates from one cluster versus all the other clusters.** (E)** Kaplan-Meier survival plots of LIHC patients (n = 371) from TCGA, the expressions of top ten genes were used to stratify patients into binary subgroups (high and low).

Next, we aimed to dissect the intratumor heterogeneity (ITH) and molecular characteristics of the various cancer cell subsets. Unsupervised clustering analysis on the 11,228 cancer cells disclosed 7 distinct clusters (C1-C7) with respective variable proportions among the patients (Figure 2A-B, S2A-D). Furthermore, large-scale copy number variations (CNVs) were inferred from the scRNA-seq data cancer cells exhibiting remarkably higher CNV levels than the referenced normal hepatocytes, confirming the identity of the cancer cells (Figure S2E). To characterize their functions in greater detail, we conducted gene set variation analysis (GSVA) analysis and observed significant phenotypic diversity (Figure 2C). Notably, cluster C6 displayed high expression of genes involved in interferon-γ/α response, IL-6/JAK/STAT pathway, angiogenesis and epithelial-mesenchymal transition (EMT), and all these are associated with tumor maintenance and metastasis.

To predict the transcription factor regulatory networks (regulons) of each cancer cell cluster, we exploited SCENIC (single-cell regulatory network inference and clustering) 16 to score the regulon activity by the AUCell algorithm (AUC score) across all single cells. By scanning and examining the transcription factor binding sites as well as the co-expression of transcription factors (TFs) and their putative target genes, we summarized the top 3 TFs from each cluster (Figure 2D) and observed the highly up-regulated NFE2L1 and NFE2L3 expression in cluster C6 as an important regulator of cancer malignancy 17-19. The results also indicated intratumor heterogeneity through diverse TF regulation.

We further utilized the liver HCC (LIHC) cohort from The Cancer Genome Atlas (TCGA) database and stratified the samples according to the top 10 cluster-specific (C1-C7) gene marker expression. Remarkably, Kaplan-Meier curves showed that high expression of the corresponding marker genes from cluster C6 was exclusively and significantly associated with poorer overall survival (OS) (Figure 2E). This finding suggests that the transcriptomics features in cluster C6 were closely related to the prognostication of HCC.

### Elucidation of stemness-related landscape and the identification of CD24/CD47/ICAM1 subclone in cancer cells

**Figure 3:**
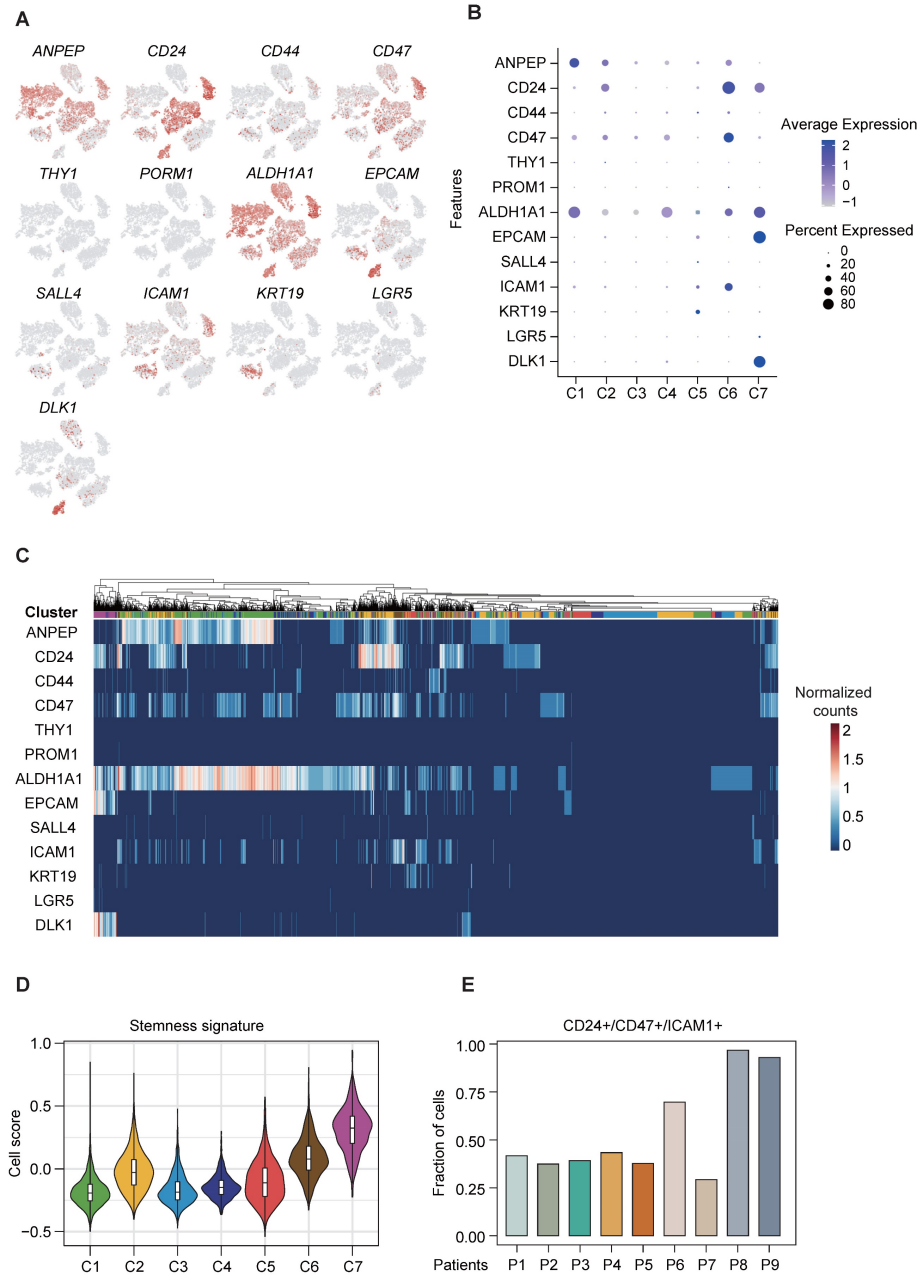
** CSC-associated heterogeneity in HCC. (A)** t-SNE plot, color-coded for expression (gray to red) of typically reported cancer stem cell markers in cancer cells.** (B)** DotPlot visualization of the relative expression of cancer stem cell markers in each subset, where the size represents the percentage of cells expressing the gene of interest and the color indicates the scaled average expression of the gene of interest across the various.** (C)** Intra-heterogeneity at the gene expression level for a panel of CSC markers.** (D)** Violin plot showing stemness signature in subpopulations of HCC. The middle box shows the median and interquartile range (IQR 25th-75th percentiles).** (E)** Bar plot showing the fraction of cells with triple expression of CD24/CD47/ICAM1 in each patient.

Cancer stemness is associated with important hallmarks of tumor behavior, including tumor initiation, metastasis, drug resistance and tumor recurrence, and tumor cells with enhanced stemness possess the stronger self-renewal ability. As the aforementioned results indicate the existence of variation in the functional and regulatory enrichment across cancer cell subpopulations (Figure 2C-D), we therefore further assessed their liver CSC marker expression, based on a panel of established liver CSC markers previously reported 5. Our results displayed disparate expression levels of these liver CSC markers across the 7 clusters of cancer cells, with some markers ubiquitously expressed in all sub-clusters and some others exclusively expressed in certain sub-clusters (Figure 3A-C). We identified the co-expression of ALDH1A1 and ANPEP in cluster C1, co-expression of CD24/CD47/ICAM1 in cluster C6, and co-expression of ALDH1A1/CD24/DLK1/EPCAM in cluster C7 (Figure 3B-C). Furthermore, clusters C6 and C7 were enriched with higher stemness signature scores 20 than the other clusters (Figure 3D), indicating their greater stemness-related potential. Notably, cluster C6 was of particular interest, given the aforementioned finding suggesting its putative stemness capability with poorer prognostication. Of note, cluster C6 cancer cells mainly came from two patients (P8 and P9), in which more than 90% of cancer cells were demarcated with triple expression of CD24/CD47/ICAM1 (Figure 3E). The presence of concurrent expression of CD24, CD47 and ICAM1 in clinical specimen was confirmed by multi-color immunofluorescence staining (Figure S3).

### *In vitro* and *in vivo* functional characterization consolidated S100A11 as a downstream stemness-related mediator

**Figure 4:**
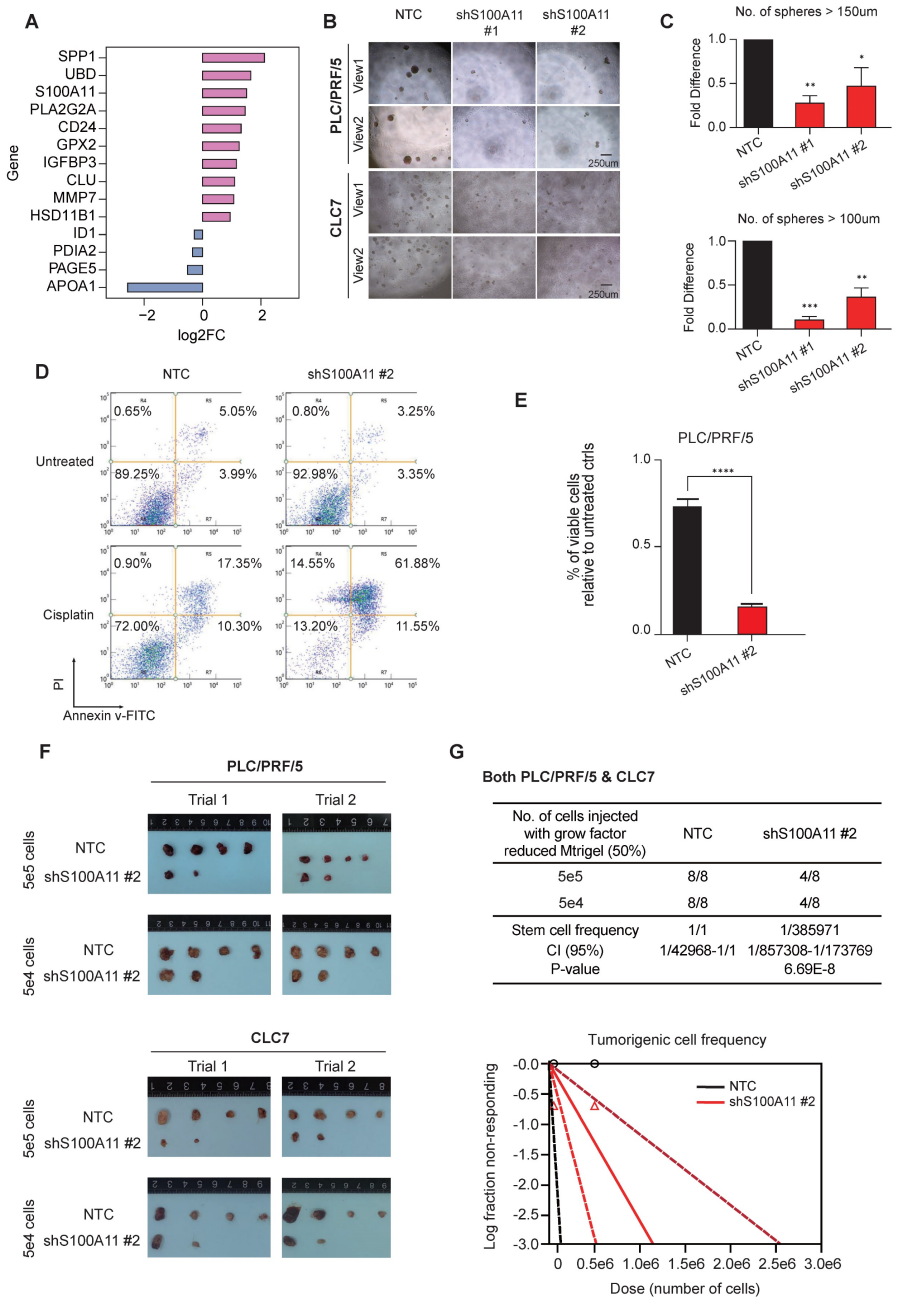
** S100A11 enhanced cancer cells stemness both *in vivo* and *vitro*. (A)** Bar plot showing the expression log2FC of up/down-regulated DEGs. Significance was determined by Wilcoxon test at* p*-value < 0.05.** (B)** and **(C)** Spheroid formation assay showed that S100A11 knockdown by two independent shRNA sequences dramatically reduced the ability of PLC/PRF/5 and CLC7 cells to form spheres compared with the non-treated control (NTC) after 10-day incubation. Scale bar, 250 µm. The data are reported as mean ± SEM (**p < 0.01).** (D)** and **(E)** S100A11 knockdown and non-target control (NTC) PLC/PRF/5 cells treated with and without cisplatin were analyzed by Annexin V assays using flow cytometry detection.** (F)** and **(G)** *In vivo* limiting dilution assays with varying numbers of NTC and S100A11-knockdown cells for both PLC/PRF/5 and CLC7 cells subcutaneously injected into NOD-SCID mice (n = 4/group), respectively, were performed. Tumor incidence was examined at day 70 post-inoculation and frequency of tumor initiating cells was calculated.

The gene expression profiles of cancer cells were comprehensively characterized and compared among the various clusters. Among the top DEGs of cluster C6 (Figure 4A), SPP1 and UBD have already been investigated in depth in previous studies 21, 22, exemplifying the potential functional roles elicited by cluster C6 cells. To this end, we shortlisted S100A11, which was the next top candidate, as our target gene for further functional interrogation with both *in vitro* and *in vivo* experiments. We adopted a stable gene knockdown approach to establish knockdown clones of S100A11 (shS100A11) in PLC/PRF/5 and CLC7 HCC cell lines and confirmed their knockdown efficiency (Figure S4). Stable knockdown shS100A11 clones showed markedly reduced sphere-forming ability (test for self-renewal ability* in vitro*) (Figure 4B-C) and chemoresistance on cisplatin (there were no significant effects on other drugs, including sorafenib, lenvatinib, 5-FU, doxorubicin and gemcitabine [data not shown]) (Figure 4D-E).

*In vivo* tumorigenicity assay by subcutaneous inoculation of shS100A11 and non-target control (NTC) HCC cells respectively in NOD-SCID mice showed that S100A11 knockdown in both HCC cell lines significantly reduced the tumor incidence (Figure 4F-G), indicating the role of S100A11 in tumor initiation and growth. Collectively, these results indicate that S100A11 is likely a downstream mediator for the stemness capacity in the cluster C6 subclone of HCC tumors.

### Reconstruction of the functional trajectory of tumor-infiltrating immune cells

**Figure 5:**
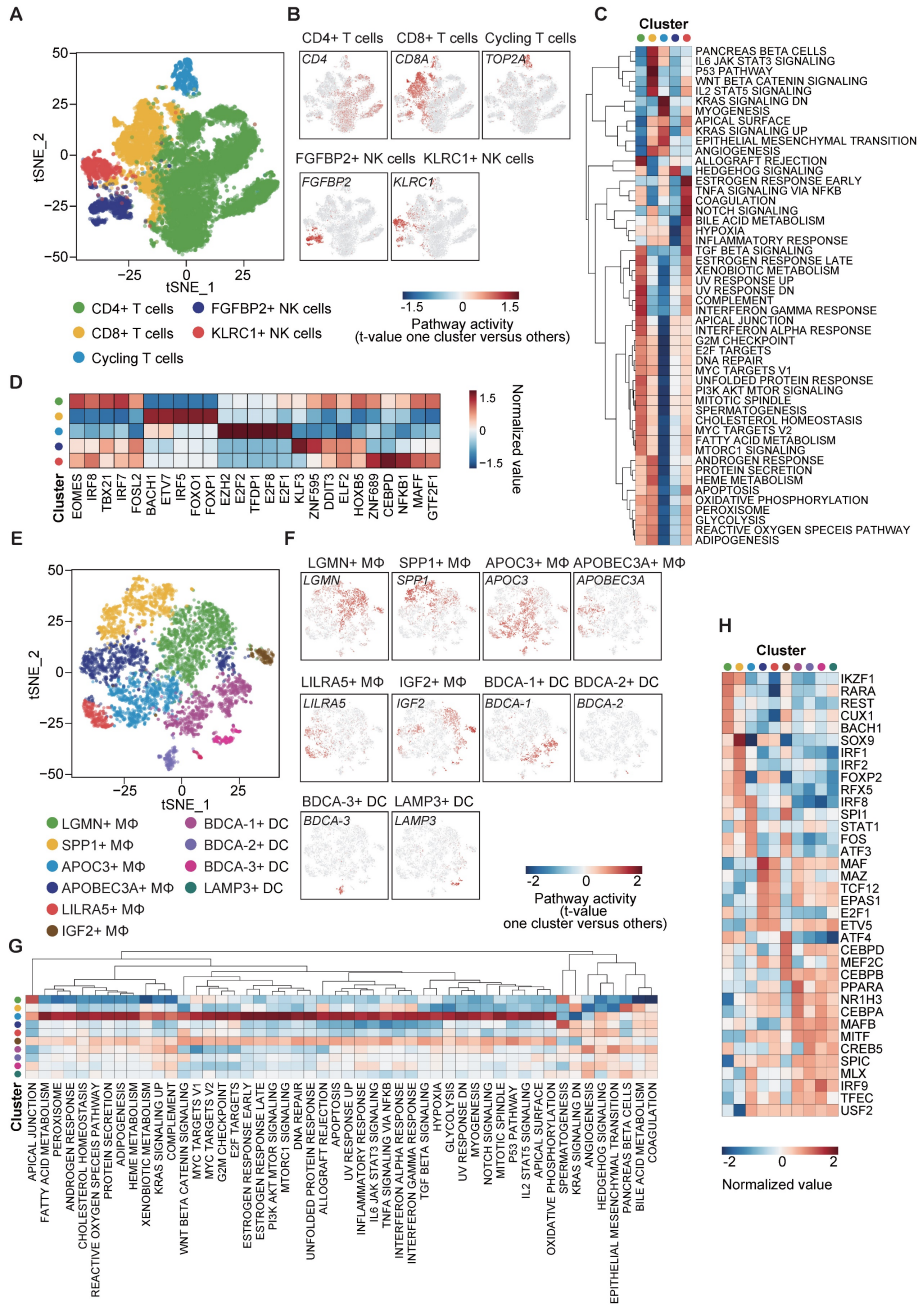
** Dissection and subclusters of lymphocytes/monocytes in HCC. (A)** t-SNE plot of lymphocytes (exclude B cells), color-coded by the subsets.** (B)** t-SNE plot, color-coded for expression (gray to red) of marker genes for the subsets as indicated.** (C)** Differential activity pathways in the five lymphocyte subsets (scored by GSVA for each cell). Red represents upregulated pathways; blue represents down-regulated pathways.** (D)** Heatmap showing the regulon activity of five lymphocyte subsets estimated by SCENIC, depicting the top five transcription factors from one cluster versus all other clusters.** (E)** t-SNE plot of monocytes, color-coded by the subsets.** (F)** t-SNE plot, color-coded for expression (gray to red) of marker genes for the subsets as indicated.** (G)** Heatmap showing the regulon activity of ten monocyte subsets estimated by SCENIC, depicting the top five transcription factors from one cluster versus all the other clusters.** (H)** Differential activity pathways in the ten monocyte subsets (scored by GSVA for each cell). Red color represents upregulated pathways; blue color represents down-regulated pathways.

To obtain a clearer understanding of the tumor immune microenvironment (TIME), we re-clustered lymphocytes and myeloid cells, respectively (B cells were excluded from the analysis due to their very limited numbers, which did not permit reliable and conclusive analysis). Firstly, 14,008 lymphocytes were clustered into 5 groups, including CD4^+^ T cells (marked with CD4 expression), CD8^+^ T cells (marked with CD8A expression), cycling T cells (marked with TOP2A expression), FGFBP2^+^ NK cells and KLRC1^+^ NK cells (Figure 5A-B). GSVA analysis for hallmark signatures demonstrated that CD4^+^ T cells were enriched in genes that are highly expressed in the allograft rejection and interferon-γ (IFN-γ) response pathways. CD8^+^ T cells had distinct expression hallmarks regarding induction of positive regulators of immune-related signatures, such as IL-6/JAK/STAT3, p53 and Wnt/β-catenin pathways, which are intricately associated with T cell proliferation and exhaustion. We also noted the enrichment of KRAS and EMT signaling pathways in cycling T cells (Figure 5C).

NK cells play a critical role in innate immunity and complement the adaptive immunity to fight against tumor 23. Here, we classified NK cells into 2 subpopulations with high expression of FGFBP2 and KLRC1, respectively (Figure 5A-B). The KLRC1^+^ NK cell subpopulation exhibited up-regulated expression of genes in the TGF-β and NOTCH signaling pathways, suggesting immune-metabolic dysfunction (Figure 5C). On the other hand, the FGFBP2^+^ NK cell subpopulation showed down-regulation of the expression of the hypoxia-related genes (Figure 5C).

In addition, subsequent TF analysis revealed top TF candidates that underlay the different lymphocyte subsets (Figure 5D). Interestingly, genes regulated by EOMES, IRF8 and IRF7 were upregulated in CD4^+^ T cells, whereas those regulated by FOXO1 and FOXP1 were upregulated in CD8^+^ T cells. Notably, EOMES and IRF8 are involved in the polarization of Th1 and Th17 cells 24 and FOXO1 has reactivation and self-renewal functions in CD8^+^ T cells 25. These results also revealed the heterogeneity of regulons across the 5 lymphocyte subpopulations (Figure 5D).

To investigate the heterogeneity landscape of myeloid cells, we stratified the 5,568 myeloid cells into 10 subsets, including 6 TAM and 4 DC subsets (Figure 5E-F). The main subsets of TAMs were distinguished by the combination of 6 markers: LGMN, SPP1, APOC3, APOBEC3A, LILRA5 and IGF2. Among these 6 markers, highly up-regulated co-expression of LGMN and SPP1 in the macrophage subpopulation has been reported in various diseases with tumor-promoting and pro-fibrotic characteristics 21, 26-28, although some conclusions remain controversial.

DCs are pivotal in orchestrating antiviral immunity. Our scRNA-seq data distinguished four subpopulations of DCs, which comprised BDCA-1 (CD1c)^+^ type 2 classical DCs (cDC2), BDCA-2 (CD303)^+^ plasmacytoid DCs (pDCs), BDCA-3 (CD141)^+^ type 1 classical DCs (cDC1) and LAMP3^+^ DCs. The former 3 DC subpopulations have been widely investigated while a recent single-cell study has revealed the existence of a previously unnoticed group of LAMP3^+^ DCs in a mature state in HCC 11. On the other hand, our GSVA results revealed a broad spectrum of expression of genes in the APOC3^+^ TAMs towards 51 hallmark pathways, suggesting a large profile of up-regulated genes in this cluster (Figure 5G). Similarly, TF analysis showed a diversity of regulons in each subset, indicating the functional discrepancy (Figure 5H).

### Bi-directional crosstalk between M2-like TAMs and cancer cells

**Figure 6:**
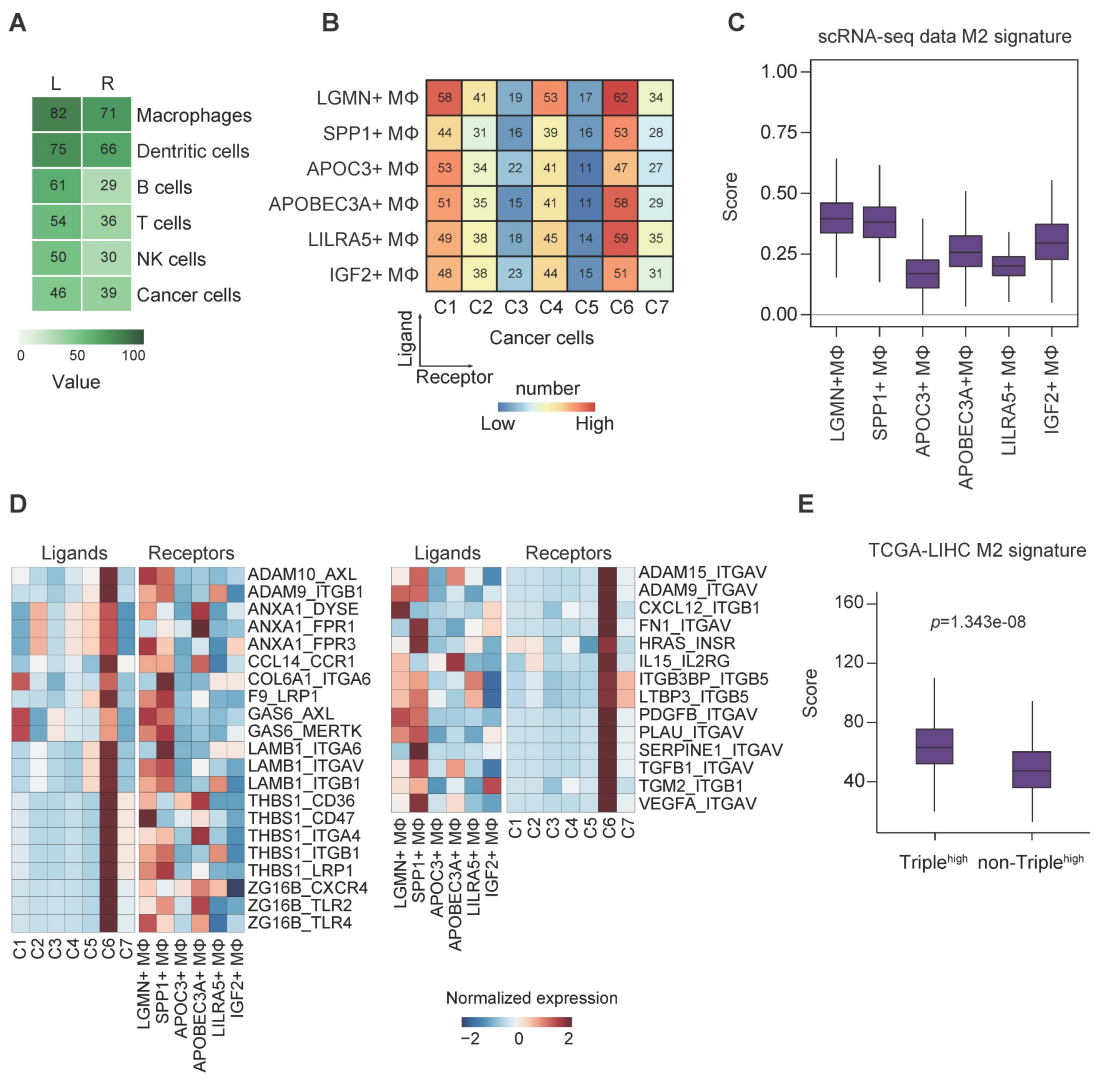
** Interrogation of cell-cell communication between TAMs and cancer cells in HCC. (A)** Heatmap showing the number of potential ligands and receptors among the cell components.** (B)** Heatmap showing the number of potential ligand-receptor pairs between TAM subsets and cancer cell subsets (TAMs as ligands source and cancer cells as receptor source).** (C)** Module scores of M2 signatures for each TAMs subpopulation (Genes list in from Azizi et al.).** (D)** Ligand-receptor pair expression according to cell type. Ligands are indicated in the left panel, and receptors are indicated in the right panel.** (E)** Module scores of M2 signatures for CD24/CD47/ICAM1high HCC tumors compared to the rest tumor samples in the TCGA-LIHC dataset. Significance was determined by Wilcoxon test at* p*-value < 0.05.

We leveraged the method proposed by Vento-Tormo et al 29 to explore the potential interaction between the various cell types. Endothelial cells (ECs) were excluded from the analysis due to their low abundance within our samples. Interestingly, we noted an exceedingly frequent communication among the immune cells, with relatively high expression of ligands and the corresponding receptors in respective cell types. Of note, we observed that TAMs had strong interactions, with contributions as both ligands and receptors source. This abundant interaction was followed by DCs and B cells, but not T cells, probably due to the exhausted state of the T cells (Figure 6A).

Given that TAMs were the most prominent ligand contributor, we set out to investigate the roles of TAMs in regulating cancer cells. Frequent connections were observed between the macrophage clusters (providing ligands) and cancer cell clusters (C6 in particular; providing receptors) (Figure 6B).

On the contrary, all the cancer cell clusters had a strong connection with many of the macrophages in general (except APOC3^+^ and IGF2^+^ TAMs) when TAMs provided receptors instead, indicating possibly less specific crosstalk in this direction (Figure S5A). To this end, we speculated a distinctive association existed between cancer stemness and TAM function in HCC. It is well accepted that TAMs can enhance cancer cell proliferation to promote tumorigenesis, and reversely, the recruitment of TAMs through the secretion of chemokines or polarization alteration by CSCs to build an immunosuppressive environment 6. Binary polarization system is commonly implicated in macrophage studies; however, in our study, TAMs showed mixed polarization states, which suggest the complexity of the residential TAM functionality (Figure S5B).

Next, we sought to derive the M1/M2 signature scores, as demonstrated in previous report 30, of the TAM subsets based on their expression of signature genes. The results showed that LGMN^+^, SPP1^+^, APOC3^+^ and IGF2^+^ TAMs were more M2-like, whereas the remaining ones were more M1-like (Figure S5C). Of note, LGMN^+^ and SPP1^+^ TAMs possessed the highest M2 signature score, supporting their putative M2 identity (Figure 6C). In addition, we also noted that LGMN^+^ and SPP1^+^ TAMs represented more than half of the TAMs (Figure S5D) and they mostly came from patients P8/P9 (Figure S5E-F), the same patients with identified cancer cell cluster C6 that displayed stemness-related phenotypes (Figure S2D).

To decipher the relationship between TAM polarization and cancer stemness, we stratified the TAMs into M2^high^ (LGMN^+^ and SPP1^+^ TAMs) and M2^low^ (the remaining TAMs) based on M2 signature scores (Figure 6C, S5C) and subsequently analyzed the cell-cell communication pinpointing ligand-receptor interactions (Figure 6D). We found 59 (31+28) unique interactions between cancer cell cluster C6 and M2^high^ cells but not between the other cancer cell clusters and M2^low^ cells (Figure S5G). There were strong connections via the GAS6-AXL/MERTK and THBS1-CD36/CD47/ITGA4/ITGB1/LRP1 axes between cancer cells (contributing ligands) and TAMs (contributing receptors) (Figure 6D, left panel). These axes were associated with immune suppression, tumor invasiveness and progression 31-33. More importantly, the findings implicate the ability of cluster C6 to drive TAM towards an M2-like phenotype 34, 35. Conversely, we examined the interaction of TAMs (contributing ligands) with cancer cells (contributing receptors) (Figure 6D, right panel). The presence of high expression of CXCL12-ITGB1 pair (Figure 6D, right panel) could promote the stem-like properties of OV6^+^ CSCs in HCC through Wnt/β-catenin pathway 36, while VEGFA-ITGAV pair may implicate the initiation and stemness in various tumors 37. We believe there exists bi-directional cross-talk between cancer cells and TAMs. To identify the potential ligand-receptor pairs that could possibly mediate M2 polarization of macrophages, we identified ligands of cancer cells (Figure 6D, left panel) and constructed a siRNA library to systematically evaluate their influence on M2 polarization upon their knockdown. After siRNA transfection, HCC cells knocked down for individual target ligands were subjected to co-culture assay with THP-1 cells. The expression of M1 markers (CD68, CD80 and CD86) and M2 markers (CD204, CD206 and CD163) in THP-1 cells were determined by qPCR. We found that upon knockdown of several ligands (GAS6, ADAM9 and ANXA1) in HCC cells, there was significant upregulation and reduction in at least one M1 and M2 markers, respectively (Figure S6A). Intriguingly, knockdown of GAS6, ADAM9 and ANXA1 could result in downregulation of S100A11 in HCC cells (Figure S7). On the other hand, to substantiate the potential ligand-receptor pairs that could possibly mediate HCC stemness, we identified ligands of macrophages (Figure 6D, right panel) and constructed another siRNA library to systematically evaluate their influence on cancer stemness upon their knockdown. After siRNA transfection, M2-differentiated THP-1 cells knocked down for individual target ligands were subjected to co-culture assay with HCC cells. The expression of liver cancer stem cell markers (ICAM1, CD47, CD24 and EPCAM) in HCC cells were determined by qPCR. We found that upon knockdown of several ligands (VEGFA, ITGB3BP and ADAM9) in M2 macrophages, there was significant reduction in at least one of the liver cancer stem cell markers tested (Figure S6B). Taken together, we believe these screenings can suggest putative mechanistic ligand-receptor pairs that could possibly drive M2 polarization of macrophages and stemness properties of HCC cells. Furthermore, GO enrichment analysis displayed a global increase of tumor-related functions when cancer cells provided the ligands, particularly in relation to functions such as cell adhesion and extracellular matrix organization, whereas phagocytosis and apoptotic cell clearance were detected in the reverse direction (Figure S5H).

In addition, since the cancer cell cluster C6 was characterized by concurrent enrichment of liver CSCs markers CD24, CD47 and ICAM1, we further sought to confirm the interaction between cluster C6 cancer cells and M2-like TAMs using TCGA-LIHC cohort. The results consistently indicate more M2-like signatures in HCC tumors with higher expression of CD24, CD47 and ICAM1 (Figure 6E), providing support to our cell-cell communication findings.

### Co-culture systems confirmed the bi-directional feedback loop between cancer stemness and TAM polarization

**Figure 7:**
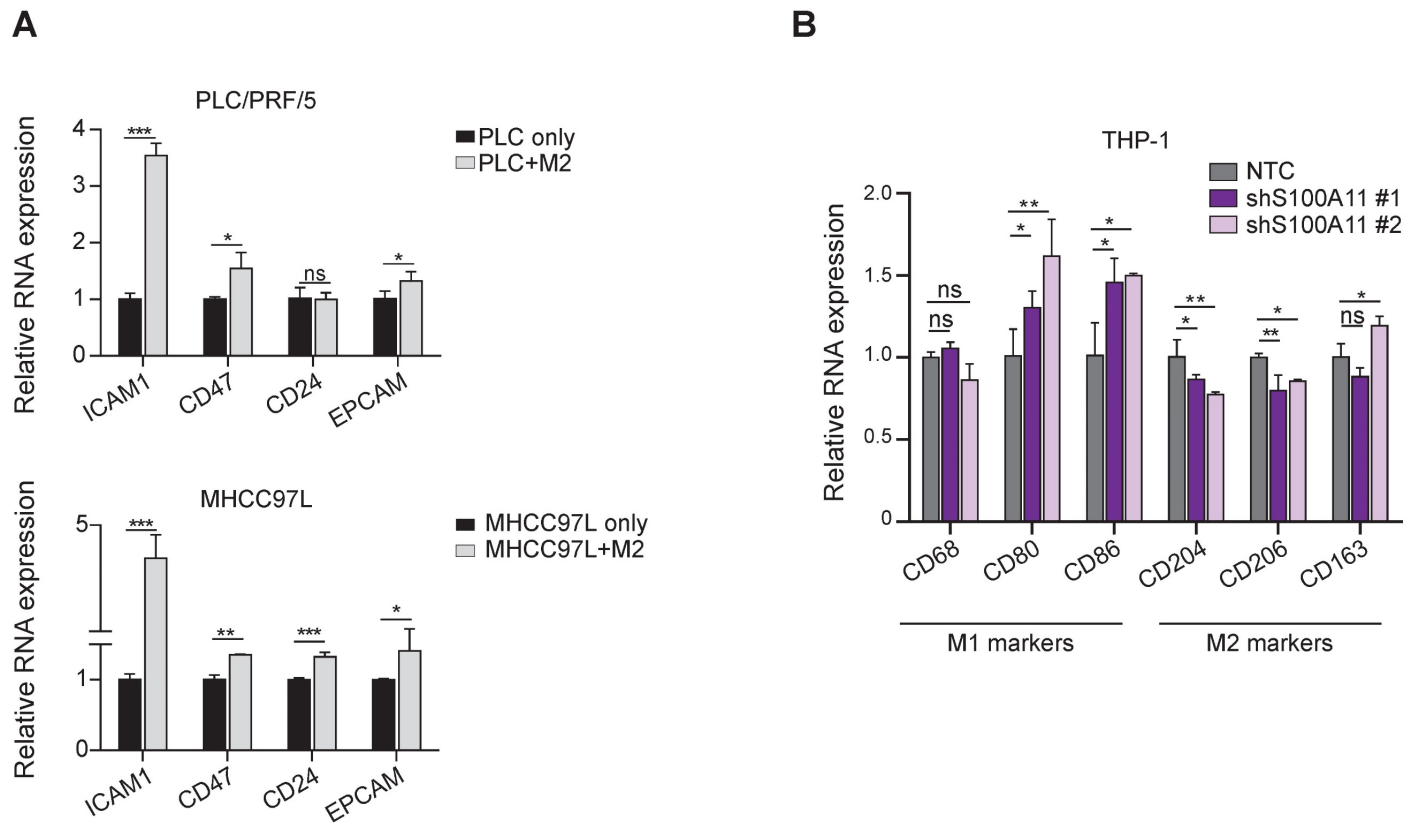
** Reciprocal interactions between TAM polarization and cancer cell stemness by co-culture assays. (A)** Barplot representing the mean expression value of the CSC markers using qPCR, normalized to three reference genes. Error bars indicate the standard error. *p < 0.05, **p < 0.01, ***p < 0.001.** (B)** Barplot representing the mean expression value of M1/M2 macrophage markers using qPCR, normalized to three reference genes. Error bars indicate the standard error. *p < 0.05, **p < 0.01, ***p < 0.001.

Intrigued by these in silico results, we used transwell co-culture models (co-culturing HCC cells with THP-1-derived macrophages) to investigate the implicated bi-directional association between cancer cell stemness and TAM polarization. The treatment with PMA (phorbol 12-myristate 13-acetate) on the THP-1-derived macrophages was aimed to generate M0 macrophages, treatment with IL-4 (interleukin-4) and IL-13 (interleukin-13) to generate M2-like macrophages, while treatment with IFN-γ (interferon-gamma) and LPS (lipopolysaccharide) was aimed to form M1-like macrophages. We observed that the M2-like TAMs upregulated the expression of CD24, CD47 and ICAM1 (markers enriched in cluster C6 cancer cells) in MHCC7L HCC cells (Figure 7A). Conversely, knockdown of S100A11 in HCC cells increased the expression of anti-cancer M1 macrophage markers CD80 and CD86 but suppressed the expression of pro-cancer M2 markers CD204 and CD206 upon co-culture with THP-1 derived macrophages. The findings suggest that S100A11 could promote macrophage polarization into M2-like phenotype to exert potential immunosuppressive roles in re-modeling the TIME to favor HCC development (Figure 7B). Moreover, to further substantiate the functional role of S100A11 in driving M2 polarization of macrophages, we established S100A11 knockdown Hepa1-6 cells and orthotopically injected them into C57BL/6 mice. There was a mild trend for reduction in tumor mass upon S100A11 knockdown. After dissociating the HCC tumor, we observed a significant increase in M1 macrophages upon S100A11 knockdown whereas the total tumor infiltrating macrophages remained unchanged (Figure S8). We also conducted co-culture-based transwell migration assays and observed that the migratory ability of M0/M1/M2 macrophages was unaffected by S100A11 knockdown (Figure S9), indicating that cluster C6 cancer cells might not likely recruit TAMs to the tumor site. Taken altogether, our cell-cell communication analysis and the subsequent functional confirmations support the bi-directional feedback loop between cancer cells cluster C6 and M2-like TAMs.

## Discussion

In this study, we employed a high-resolution single-cell approach to characterize the tumor and immune components within the TIME, with a particular focus on cancer stemness and cell-cell communication. We provided a detailed distribution of CSC-related markers at cellular resolution across the patients' tumors. With this, we identified a subgroup of HCC cells with concurrent expression of CD24, CD47 and ICAM1, which correlated with stemness characteristics and poorer survival. We also discovered a mutual regulation between stemness property and macrophage polarization, specifically M2-like macrophages.

To the best of our knowledge, the concurrent expression of CD24, CD47 and ICAM1 has not been reported in HCC. Our study also revealed the novel downstream mediator S100A11, and its downregulation resulted in reduced cancer stemness properties. The S100 protein family has been implicated in hepatic steatosis, non-alcoholic steatohepatitis, and HCC 38. Specifically, S100A11 is involved in cell proliferation, invasion, endoplasmic reticulum stress and drug resistance. A recent study by Sobolewski et al 39 utilized a proteomic approach and suggested that S100A11/ANXA2 overexpression was a hallmark of liver inflammation/fibrosis and associated with high-grade HCC and poor clinical outcomes. Additionally, S100A11 was also implicated in de-differentiation and EMT of hepatocytes. Our findings provide new insights into the role of S100A11 in HCC pathogenesis, suggesting its potential as a therapeutic target for further study.

The interplay among the diverse cell types in the TME is crucial in tumor progression and therapeutic outcomes. Our study has unraveled a unique association between LGMN/SPP1^+^ TAMs and stemness-related cancer cells (cancer cell cluster C6 marked by co-expression of CD24, CD47 and ICAM1). Our further investigation unveiled a reciprocal regulation between TAM polarization and cancer cell stemness.

With GSVA analysis, we identified a list of up-regulated genes within the hallmark gene sets in CD4^+^ T cells and KLRC1^+^ (NKG2A) NK cells. As a subtype of immunosuppressive NK cells, we observed a high level of TGF-β signaling in KLRC1^+^ NK cells (Figure 5C), suggesting its negative impact on anti-tumor immunity, particularly on CD8^+^ T cells. In general, TGF-β is a crucial cytokine that can directly inhibit CD8^+^ T cell cytotoxicity, stimulate the generation of regulatory T cells, and contribute to the exclusion of T cells from tumor core. Furthermore, it can promote the immunosuppressive TIME that favors evasion of tumor surveillance 23. Our analysis also revealed an enrichment of IL-6/JAK/STAT3 signaling in CD8^+^ T cells, which is known to enhance proliferation, survival, invasiveness, and metastasis of tumor cells 40. The various immunosuppressive factors induced by individual malignant and non-malignant components of the TIME may contribute to the exhausted T cell phenotype to evade immune surveillance.

Additionally, the distinct regulatory networks of the TFs within the sub-clusters also suggested their potentially diverse functions. For example, lines of evidence have suggested that EOMES^+^ CD4 T cells, which we observed in this study, tend to accumulate in inflamed tissues and may play a role in chronic inflammatory disorders 41. We also found the high expression of FOXO1 in CD8^+^ T cells, a control point for the reactivation and self-renewal 25. With reference to myeloid cells and consistent with previous reports 26, 27, we identified LGMN^+^ and SPP1^+^ TAM clusters in our data, with a particular association with cancer cells. In addition, we also demonstrated the existence of LAMP3^+^ DCs, which play a pivotal role in regulating the function of lymphocytes and recruiting CD4^+^ T cells 11.

Taken together, this study provided insights on the presence of a cancer-stemness subpopulation of HCC cells with S100A11 as its downstream mediator. We also demonstrated the presence of a bi-directional crosstalk between this subpopulation of HCC cells and TAMs, resulting in the maintenance of the expression of cancer stem cell markers and driving M2-like TAM polarization towards a pro-tumorigenic niche. We hope our findings will lead to a better understanding of HCC progression and help to identify new therapeutic targets.

## Materials and methods

### Sample collection

We obtained informed consent from all patients. Of note, 5 of them were previously reported 14. Samples were selected randomly, which complied with the criteria of HBV infection and having more than 1000 viable single cells from dissociated tumor cell suspension per case. HCC tissues were obtained intraoperatively from patients who underwent surgical resections at the Queen Mary Hospital and Queen Elizabeth Hospital of Hong Kong. All protocols and experiments were approved by the Institutional Review Board of the University of Hong Kong/Hospital Authority Hong Kong West Cluster (UW 17-056).

### Tissue dissociation

We followed the tissue dissociation and viable singlet cell selection protocols as we previously established 14. Finally, Trypan blue staining was carried out for cell concentration evaluation. The viable singlet cell suspension was prepared to the desired concentration range (100-2000 cells/µl), as recommended by the user protocol of 10X Genomics.

### Library preparation and sequencing

To perform scRNA-seq experiments, the Chromium platform (10X Genomics, California, USA) was used. The platform contains microfluidics circuits to allow droplet-based capture of single cells together with barcoded oligos on gel beads in oil emulsion droplets. Sequencing libraries were subsequently constructed as described 14. The sequencing libraries were then subjected to the Illumina Novaseq platform and we ensured they were sequenced with adequate coverage.

### Cell culture

PLC/PRF/5 (CRL-8024) was obtained from American Type Culture Collection (ATCC). CLC7 was a kind gift from Dr. Lijian Hui of Shanghai Institutes for Biological Sciences. HCC cell lines used in this study were authenticated by short tandem repeat (STR) DNA Profiling and no cellular cross-contamination (Figure S10) or mycoplasma contamination was detected. PLC/PRF/5 cell line was cultured in Dulbecco's Modified Eagle Medium (DMEM-HG, Invitrogen Gibco, Massachusetts, USA) supplemented with 10% (v/v) fetal bovine serum (FBS), 1 × (50 unit/ml) penicillin-streptomycin cocktail (P/S) (Gibco, USA). CLC7 cells were cultured in the RPMI1640 medium (Invitrogen Gibco, Massachusetts, USA) supplemented with human recombinant EGF (Thermo Fisher PeproTech, Massachusetts, USA) and ITS-A (Invitrogen Gibco, Massachusetts, USA). Cell lines were cultured at 37°C and 5% CO_2_ incubator.

### Establishment of stable knockdown and spheroid formation assay

Stable knockdown cells were established as previously described 42, 43. Oligos for short hairpin RNAs (shRNAs) targeting the various gene mRNA were subcloned into pLKO.1-Puro shRNA expression vector. The shRNA sequences were listed in supplementary Table S2. Viral packaging for the lentiviruses containing the shRNA vectors was carried out in HEK293FT according to the manufacturer's protocol for the MISSION® Lentiviral Packaging System (Sigma-Aldrich, St Louis, MO). Then, the viral particles containing shRNA were transduced into HCC cells to establish the shRNA stably expressing cells. Total RNA was extracted by Trizol (Invitrogen) and cDNA was synthesized by reverse transcription kit (Invitrogen). Western blotting using anti-S100A11 antibody (Proteintech (10237-1-AP), IL, USA) was performed to validate the reduction of protein levels in the respective knockdown cells.

For the spheroid formation assay 44, 45, cells were cultured in 0.25% methylcellulose in serum-free DMEM/F12 in wells pre-coated with 1% polyHEMA in the presence of B-27 supplement to allow spheroid formation. At end point, the numbers of spheres greater than 100μm in diameter were counted. Experiments were done thrice independently.

### Annexin V assay for drug resistance

Cells were treated with or without 0.8µg/ml cisplatin in the respective media for 48h for PLC/PRF/5 and CLC7 cell lines. The culturing media were kept and the attached cells were trypsinized after PBS washing. The trypsinized cells had trypsin neutralized by the FBS-containing media which were mixed with the above kept media. Cells were washed once in PBS after centrifugation at ~1400g for 5min. Commercial kit for annexin V assays (BD Biosciences, Sparks, MD) was used according to the manufacturer's instructions. By using a ZE5 flow cytometer (Bio-rad, and CellQuest software (BD Biosciences), the percentages of apoptotic cells were determined under proper gating for PI and annexin V signals as described 46.

### Tumorigenicity assay

For the subcutaneous tumorigenicity models 47, 5×10^4^ or 5×10^5^ viable HCC cells (PLC/PRF/5 and CLC7, each with both shS100A11 and NTC) were re-suspended in 100ul 1:1 ice-cold DMEM medium-Matrigel mix (v/v) and subcutaneously injected into 6-week-old, male, immunocompromised NOD-SCID mice. The size of the tumor was monitored over a period of 3-5 (PLC/PRF/5) or 8-10 (CLC7) weeks. The tumor mass (g) of the harvested tumor was measured at the endpoint of the experiment. The tumor volume in mm3 was calculated by the following formula: 1/2 × the longer diameter (mm) × power 2 of the shorter diameter (mm). The animal experiment was approved by the Committee on the Use of Live Animals in Teaching and Research (CULATR 5688-21), Li Ka Shing Faculty of Medicine, University of Hong Kong.

### Orthotopic injection assay

Orthotopic liver injection model was performed to investigate the tumor growth and tumor-infiltrating macrphages. Briefly, 3.5×10^6^ mouse hepatoma Hepa1-6 cells (NTC and shS100a11) were injected into the left lobes of livers of C57BL/6 mice. Each experimental group had at least 6 mice. After 2 weeks, the mice were sacrificed. Tumor mass was recorded. Tumors from C57BL/6 mice were dissociated for detecting tumor infiltrating macrophages by flow cytometry.

### Transwell co-culture assays

To investigate the effect of tumor associated-macrophages on HCC stemness, HCC/THP-1 co-culture assay was utilized. 1×10^5^ HCC cells were seeded in upper chamber (0.4μm pore size, 12 mm diameter; MilliporeSigma, Massachusetts, USA). M0, M1 or M2-differentiated THP-1 cells were seeded in the lower chamber containing 750 μl RPMI-1640 medium with 10% FBS. After 24-72 hours of co-culture, HCC cells were harvested. RNA extraction and reverse transcription (RT) were conducted to generate cDNA. Expression of stemness markers, including ICAM1, CD47, CD24 and EPCAM were determined by quantitative real-time PCR (qRT-PCR).

To evaluate the effect of S100A11 on macrophage polarization, 1×10^5^ PLC/PRF/5 cells (NTC, shS100A11) were seeded in the upper chamber (0.4μm pore size, 12 mm diameter; MilliporeSigma, Massachusetts, USA). The lower chamber contained 2×10^5^ M0-differentiated THP-1 cells in 750 μl RPMI-1640 medium with 10% FBS. RNA was extracted from THP-1 cells after 48 hours of co-culture. Gene expression of M1 and M2 markers (CD68, CD80, CD86, CD204, CD206 and CD163) was determined by qRT-PCR.

To study the effect of S100A11 on macrophage recruitment, 2×10^5^ M0, M1 and M2-differentiated THP-1 cells were seeded in the upper chamber (8μm pore size; MilliporeSigma, Massachusetts, USA). 2×10^4^ PLC/PRF/5 cells (NTC, shS100A11) were seeded in the lower chamber. After 72 hours, the THP-1 cells in the upper chamber were fixed in methanol and underwent crystal violet staining. The cells passing through the upper chamber were counted by software Image J (National Institutes of Health, Bethesda, USA).

To identify the potential ligands that mediated HCC cell stemness and macrophage M2-polarization, we chose 9 ligands from HCC tumors and 12 ligands from macrophages by expression level in our scRNA-seq data. A small pooled siRNA library (LP_162028; Horizon Discovery Cherry-Pick Custom Library Tool, UK) was utilized to target and knockdown the ligands in the corresponding cells. Co-culture assay was conducted after 48hrs of siRNA transfection. The expression of liver CSC markers (ICAM1, CD47, CD24 and EPCAM) in HCC cells and the M1/M2 markers in macrophages were determined by qPCR.

### Quantitative Real-time PCR (qRT-PCR)

qRT-PCR was performed as described 48, 49, using ABI Power SYBR® Green master mix and detected by ABI QuantStudio 5 Real-Time PCR System (Applied Biosystems, Foster City, CA). The primer sequences are listed in supplementary Table S3.

### Multicolor immunofluorescence staining

We used Opal Polaris 7 Color Kit (NEL861001KT) according to the manufacturer's recommendation, with anti-CD24 (Abcam, Ab202073, 1:25), anti-CD47 (Abcam, Ab284132, 1:100) and anti-ICAM1 (Abcam, Ab282575, 1:200) antibodies.

### Single-cell RNA-seq data processing

The sequenced data were processed by the Cell Ranger Software Suite (v5.0.1, 10X Genomics). Briefly, Illumina base call (BCL) sequence files were demultiplexed into FASTQ format, then FASTQ files from each sample were aligned to the human GRCh38 reference genome. After the default filtering for quality control of each sample, unique molecular identifier (UMI) count matrix with feature barcode was generated for subsequent analysis.

### Dimension reduction and unsupervised clustering

The UMI count matrix was converted to Seurat object using the R package Seurat (v2.3.4) 50. Briefly, we utilized the following procedure to exclude low-quality cells of each sample: cells with fewer than 500 genes or more than 4,000 genes detected and cells for which more than 10% of UMIs were derived from mitochondrial genes were excluded. The filtered gene expression matrix was normalized, in which the number of UMIs of each gene was divided by the sum of the total UMIs per cell, multiplied by 10,000, and then transformed to log-scale. We included transcripts of variably expressed genes (top 2000 features) using the vst method for downstream analysis. Since single-cell capture may be contaminated by doublets, the R package DoubletFinder (v2.0.3) 51 was exploited to detect potential doublets. After implementing the above procedures, datasets were integrated at normalized count level across patients for better elimination of batch effect. Next, we applied PCA after standard preprocessing linear transformation ('scaling') for the integrated data. The top 40 informative PC dimensions were selected based on the k-nearest neighbor graph, which came from the Euclidean distance in PCA space and we clustered cells using the Louvain algorithm with resolution 1.5 for t-SNE. Finally, we yielded 44 different initial clusters.

### Differentially expressed gene analysis and major cell clusters identification

To characterize each cluster, DEGs of each subset were identified by the non-parametric Wilcoxon Rank Sum test via the FindMarkers function in the Seurat package and ranked by average log2FC (fold change) and *p*-value. We filtered marker genes using a minimum log2FC of 0.25 and a maximum *p*-value of 0.05. The marker genes assigned to label each cluster also generally had expression levels higher than those of the genes in the other developmentally or functionally related clusters. Seven major clusters were identified using canonical marker genes in the two-dimensional t-SNE map.

### Copy Number Variation (CNV) calling

Computationally inferring large-scale chromosomal CNVs in every cell enables us to identify the cancer cells. We used InferCNV (https://github.com/broadinstitute/inferCNV) (v1.2.1) to determine CNV profiles on single cells. We provided raw UMI count data and used the recommended parameter settings with cut-off value equal to 0.1 for the minimum average read count per gene among reference cells. We used PTPRC-negative clusters as input and the results were referenced to the normal hepatocytes from public data 52. The per-gene copy number scores calculated for each cell were visualized through heatmap.

### Sub-clustering of cancer cells and immune cells

The dimensional reduction was specifically re-performed on the cancer cells. Similar to the above cell type clustering, sub-clustering analysis of the cancer cells was performed. Clusters were then merged into major cell classes by combining related/highly similar clusters according to the expression of marker genes. Regarding the immune cells, we first stratified them according to myeloid and lymphoid origins, which were then subclustered separately. We also re-applied t-SNE dimension reduction and marker gene detection, similar to those performed on cancer cells.

### Survival Analysis

A total of 377 LIHC samples were retrieved from TCGA by using the R packages RTCGA clinical (v20151101.6.0) and RTCGA rnaseq (v1.60.0), from which we extracted the gene expression and clinical information. Survival data was extracted using the survivalTCGA function, and only 371 samples with survival data remained. Then, the TCGA samples were simultaneously split into two groups (expression “low” and “high” groups) based on the median expression of the top 10 highly expressed genes for each of the 7 major cancer cell classes. Finally, comparisons of the overall survival between expression “low” and “high” groups were performed and we generated Kaplan-Meier plots with two strata by utilizing the survfit and Surv functions of the survival package (v3.2.7) and ggsurvplot of the survminer package (v0.4.3).

### Functional enrichment analysis

Pathway analyses were predominantly performed on the 50 hallmark pathways (msigdbr package, v7.2) as described in the molecular signature database. Next, the GSVA package (v1.34.0) was applied and pathway scores were calculated for different cell clusters using the gsva function. Student's t-test was applied to identify the significantly enriched signaling pathways with *p*-value < 0.05.

### SCENIC analysis

We employed SCENIC to analyze the activity of TFs towards different cell types, which could group the target genes and the corresponding TFs into regulons and computes the activity of the regulons as the relative rank-sum of the expression of these targets (pySCENIC, version 0.9.15) 16. In brief, normalized expression matrices were used as input to generate gene regulatory networks by GRNboost2 (arboreto 0.1.5). Then, regulatory features 10 kb centered from the TSS of the cisTarget human motif database v9 were utilized to identify the enriched motifs via “ctx” function and individual cells were scored for motifs using the “aucell” function. Subsequently, Student's t-test was used to determine the significantly enriched TFs dominant in each subset.

### Cell-cell interaction analysis

Cell-cell communication networks were investigated via ligand-receptor interactions and we employed the similar analysis proposed by Vento-Tormo et al 29. Briefly, Normalized gene expression from both immune and cancer cells was taken to perform cell-cell interaction analysis based on a public repository of ligands and receptors 29, 53. All ligand-receptor pairs expressed in less than 10% of all cells within each cell type population were eliminated. A null-distribution-based permutation test was applied to identify particular and unbiased interactions on the corresponding expression of ligand-receptor partner (1000 permutations) and ligand from one cell type and the corresponding receptor from another cell type were tested with *p*-value < 0.05 was considered statistically significant.

### Definition of cell scores and signature

Gene module scores for different malignant subclusters were generated using the AddModuleScore function in Seurat (v2.3.4) 50 with normalized matrix. Gene modules were visualized using the VlnPlot function.

### Statistical analysis

For the scRNA-seq data, statistical analyses and graphics production were performed using R v3.6.3 (Foundation for Statistical Computing). For the experimental data, statistical analyses and graphics production were performed using GraphPad Prism 7 (GraphPad Software).

## Supplementary Material

Supplementary figures and tables.

## Abbreviations

HCC: hepatocellular carcinoma
CSCs: cancer stem cells
TAMs: tumor-associated macrophages
NAFLD: non-alcoholic fatty liver disease
TKI: tyrosine kinase inhibitors
ICI: immune checkpoint inhibitors
CD133: prominin 1
CD44: CD44 molecule
EpCAM: epithelial cell adhesion molecule
CD90: Thy-1 cell surface antigen
CD13: alanyl aminopeptidase
scRNA-seq: single-cell RNA sequencing
UMIs: unique molecular identifiers
PCA: principal component analysis
tSNE: t-distributed stochastic neighbor embedding
DEGs: differentially expressed genes
CD45: protein tyrosine phosphatase receptor type C
CD3D: CD3 delta subunit of T-cell receptor complex
CD3E: CD3 epsilon subunit of T-cell receptor complex
GNLY: granulysin
KLRF1: killer cell lectin like receptor F1
CD79A: CD79a molecule
MZB1: marginal zone B and B1 cell specific protein
CD14: CD14 molecule
CYBB: cytochrome b-245 beta chain
CD1C: CD1c molecule
FCER1A: Fc epsilon receptor Ia
VWF: von Willebrand factor
ENG: endoglin
GPC3: glypican 3
ASS1: argininosuccinate synthase 1
NK: natural killer
DCs: dendritic cells
ITH: intratumor heterogeneity
CNVs: copy number variations
SCENIC: single-cell regulatory network inference and clustering
TFs: transcription factors
OS: overall survival
LIHC: the liver hepatocellular carcinoma
TCGA: The Cancer Genome Atlas
ALDH1A1: aldehyde dehydrogenase 1 family member A1
CD24: CD24 molecule
CD47: CD47 molecule
ICAM1: intercellular adhesion molecule 1
DLK1: delta like non-canonical Notch ligand 1
SPP1: secreted phosphoprotein 1
UBD: ubiquitin D
S100A11: S100 calcium binding protein A11
CD4: CD4 molecule
CD8A: CD8 subunit alpha
TOP2A: DNA topoisomerase II alpha
FGFBP2: fibroblast growth factor binding protein 2
KLRC1: killer cell lectin like receptor C1
LGMN: legumain
APOC3: apolipoprotein C3
APOBEC3A: apolipoprotein B mRNA editing enzyme catalytic subunit 3A
LILRA5: leukocyte immunoglobulin like receptor A5
IGF2: insulin like growth factor 2
TIME: tumor immune microenvironment
GSVA: gene set variation analysis
IFN-γ: Interferon Gamma
BDCA-1(CD1c): CD1c molecule
cDC2: type 2 classical DCs
BDCA-2 (CD303): C-type lectin domain family 4 member C
pDCs: plasmacytoid DCs
BDCA-3 (CD141): thrombomodulin
cDC1: type 1 classical DCs
LAMP3: lysosomal associated membrane protein 3
ECs: Endothelial cells
AXL: AXL receptor tyrosine kinase
MERTK: MER proto-oncogene, tyrosine kinase
THBS1: thrombospondin 1
CD36: CD36 molecule
ITGA4: integrin subunit alpha 4
ITGB1: integrin subunit beta 1
LRP1: LDL receptor related protein 1
CXCL12: C-X-C motif chemokine ligand 12
VEGFA: vascular endothelial growth factor A
ITGAV: integrin subunit alpha V
NRP1: neuropilin 1
CD80: CD80 molecule
CD86: CD86 molecule
CD204: macrophage scavenger receptor 1
CD206: mannose receptor C-type 1
TME: the tumor microenvironment
PMA: phorbol 12-myristate 13-acetate
IL-4: interleukin-4
IL-13: interleukin-13
IFN-γ: interferon-gamma
LPS: lipopolysaccharide
shRNAs: short hairpin RNAs
RT-qPCR: real-time polymerase chain reaction
EOMES: eomesodermin
ETV7: ETS variant transcription factor 7
ATCC: American Type Culture Collection
STR: short tandem repeat
DMEM: Dulbecco's Modified Eagle Medium
FBS: fetal bovine serum

## References

[1] Llovet JM, Kelley RK, Villanueva A, Singal AG, Pikarsky E, Roayaie S (2021). Hepatocellular carcinoma. Nature reviews Disease primers.

[2] Villanueva A (2019). Hepatocellular Carcinoma. N Engl J Med.

[3] Lee J, Ng K, Wong L, Ang A, Tan SH, Tai D (2019). Immune checkpoint inhibitors and tyrosine kinase inhibitors in patients with advanced hepatocellular carcinoma: Does the sequence matter?. Ann Oncol.

[4] Tirino V, Desiderio V, Paino F, De Rosa A, Papaccio F, La Noce M (2013). Cancer stem cells in solid tumors: an overview and new approaches for their isolation and characterization. FASEB J.

[5] Lee TK, Guan XY, Ma S (2022). Cancer stem cells in hepatocellular carcinoma - from origin to clinical implications. Nat Rev Gastroenterol Hepatol.

[6] Allavena P, Digifico E, Belgiovine C (2021). Macrophages and cancer stem cells: a malevolent alliance. Mol Med.

[7] Wang YF, Yuan SX, Jiang H, Li ZX, Yin HZ, Tan J (2022). Spatial maps of hepatocellular carcinoma transcriptomes reveal spatial expression patterns in tumor immune microenvironment. Theranostics.

[8] Arvanitakis K, Koletsa T, Mitroulis I, Germanidis G (2022). Tumor-Associated Macrophages in Hepatocellular Carcinoma Pathogenesis, Prognosis and Therapy. Cancers.

[9] Arvanitakis K, Mitroulis I, Chatzigeorgiou A, Elefsiniotis I, Germanidis G (2023). The Liver Cancer Immune Microenvironment: Emerging Concepts for Myeloid Cell Profiling with Diagnostic and Therapeutic Implications. Cancers.

[10] Zhang QY, Ho DW, Tsui YM, Ng IO (2022). Single-Cell Transcriptomics of Liver Cancer: Hype or Insights?. Cell Mol Gastroenterol Hepatol.

[11] Zhang Q, He Y, Luo N, Patel SJ, Han Y, Gao R (2019). Landscape and Dynamics of Single Immune Cells in Hepatocellular Carcinoma. Cell.

[12] Sun Y, Wu L, Zhong Y, Zhou K, Hou Y, Wang Z (2021). Single-cell landscape of the ecosystem in early-relapse hepatocellular carcinoma. Cell.

[13] Zheng C, Zheng L, Yoo JK, Guo H, Zhang Y, Guo X (2017). Landscape of Infiltrating T Cells in Liver Cancer Revealed by Single-Cell Sequencing. Cell.

[14] Ho DWH, Tsui YM, Chan LK, Sze KMF, Zhang X, Cheu JWS (2021). Single-cell RNA sequencing shows the immunosuppressive landscape and tumor heterogeneity of HBV-associated hepatocellular carcinoma. Nature Communications.

[15] Ho DWH, Tsui YM, Sze KMF, Chan LK, Cheung TT, Lee E (2019). Single-cell transcriptomics reveals the landscape of intra-tumoral heterogeneity and sternness-related subpopulations in liver cancer. Cancer Lett.

[16] Aibar S, Gonzalez-Blas CB, Moerman T, Huynh-Thu VA, Imrichova H, Hulselmans G (2017). SCENIC: single-cell regulatory network inference and clustering. Nat Methods.

[17] Kobayashi A (2020). Roles of NRF3 in the Hallmarks of Cancer: Proteasomal Inactivation of Tumor Suppressors. Cancers.

[18] Kobayashi A, Waku T (2020). New addiction to the NRF2-related factor NRF3 in cancer cells: Ubiquitin-independent proteolysis through the 20S proteasome. Cancer science.

[19] Qiu L, Yang Q, Zhao W, Xing Y, Li P, Zhou X (2022). Dysfunction of the energy sensor NFE2L1 triggers uncontrollable AMPK signaling and glucose metabolism reprogramming. Cell death & disease.

[20] Phillips D, Matusiak M, Gutierrez BR, Bhate SS, Barlow GL, Jiang S (2021). Immune cell topography predicts response to PD-1 blockade in cutaneous T cell lymphoma. Nat Commun.

[21] Liu LL, Zhang RY, Deng JW, Dai XM, Zhu XD, Fu QH (2022). Construction of TME and Identification of crosstalk between malignant cells and macrophages by SPP1 in hepatocellular carcinoma. Cancer Immunol Immun.

[22] Ma L, Wang L, Khatib SA, Chang CW, Heinrich S, Dominguez DA (2021). Single-cell atlas of tumor cell evolution in response to therapy in hepatocellular carcinoma and intrahepatic cholangiocarcinoma. J Hepatol.

[23] Demaria O, Cornen S, Daeron M, Morel Y, Medzhitov R, Vivier E (2019). Harnessing innate immunity in cancer therapy. Nature.

[24] Renoux F, Stellato M, Vogetseder A, Huang R, Subramaniam A, Blyszczuk P (2019). SAT0001 FOSL-2 IS A REPRESSOR OF FOXP3 EXPRESSION DURING TREG DEVELOPMENT AND CONTROLS AUTOIMMUNITY. Ann Rheum Dis.

[25] Marcel N, Hedrick SM (2020). A key control point in the T cell response to chronic infection and neoplasia: FOXO1. Curr Opin Immunol.

[26] Wendisch D, Dietrich O, Mari T, von Stillfried S, Ibarra IL, Mittermaier M (2021). SARS-CoV-2 infection triggers profibrotic macrophage responses and lung fibrosis. Cell.

[27] Wang HB, Chen BH, Lin YY, Zhou Y, Li XB (2020). Legumain Promotes Gastric Cancer Progression Through Tumor-associated Macrophages In vitro and In vivo. Int J Biol Sci.

[28] Sun SG, Guo JJ, Qu XY, Tang XY, Lin YY, Hua KQ (2022). The extracellular vesicular pseudogene LGMNP1 induces M2-like macrophage polarization by upregulating LGMN and serves as a novel promising predictive biomarker for ovarian endometriosis recurrence. Hum Reprod.

[29] Vento-Tormo R, Efremova M, Botting RA, Turco MY, Vento-Tormo M, Meyer KB (2018). Single-cell reconstruction of the early maternal-fetal interface in humans. Nature.

[30] Filbin MG, Tirosh I, Hovestadt V, Shaw ML, Escalante LE, Mathewson ND (2018). Developmental and oncogenic programs in H3K27M gliomas dissected by single-cell RNA-seq. Science.

[31] Akalu YT, Rothlin CV, Ghosh S (2017). TAM receptor tyrosine kinases as emerging targets of innate immune checkpoint blockade for cancer therapy. Immunol Rev.

[32] Pinato DJ, Brown MW, Trousil S, Aboagye EO, Beaumont J, Zhang H (2019). Integrated analysis of multiple receptor tyrosine kinases identifies Axl as a therapeutic target and mediator of resistance to sorafenib in hepatocellular carcinoma. Br J Cancer.

[33] Poon RT, Chung KK, Cheung ST, Lau CP, Tong SW, Leung KL (2004). Clinical significance of thrombospondin 1 expression in hepatocellular carcinoma. Clin Cancer Res.

[34] Chen XC, Yang B, Tian J, Hong H, Du Y, Li K (2018). Dental Follicle Stem Cells Ameliorate Lipopolysaccharide-Induced Inflammation by Secreting TGF-beta 3 and TSP-1 to Elicit Macrophage M2 Polarization. Cell Physiol Biochem.

[35] Shibata T, Makino A, Ogata R, Nakamura S, Ito T, Nagata K (2020). Respiratory syncytial virus infection exacerbates pneumococcal pneumonia via Gas6/Axl-mediated macrophage polarization. J Clin Invest.

[36] Wang C, Wang MD, Cheng P, Huang H, Dong W, Zhang WW (2017). Hepatitis B virus X protein promotes the stem-like properties of OV6(+) cancer cells in hepatocellular carcinoma. Cell Death Dis.

[37] Beck B, Driessens G, Goossens S, Youssef KK, Kuchnio A, Caauwe A (2011). A vascular niche and a VEGF-Nrp1 loop regulate the initiation and stemness of skin tumours. Nature.

[38] Delangre E, Oppliger E, Berkcan S, Gjorgjieva M, de Sousa MC, Foti M (2022). S100 Proteins in Fatty Liver Disease and Hepatocellular Carcinoma. Int J Mol Sci.

[39] Sobolewski C, Abegg D, Berthou F, Dolicka D, Calo N, Sempoux C (2020). S100A11/ANXA2 belongs to a tumour suppressor/oncogene network deregulated early with steatosis and involved in inflammation and hepatocellular carcinoma development. Gut.

[40] Johnson DE, O'Keefe RA, Grandis JR (2018). Targeting the IL-6/JAK/STAT3 signalling axis in cancer. Nat Rev Clin Oncol.

[41] Dejean AS, Joulia E, Walzer T (2019). The role of Eomes in human CD4 T cell differentiation: A question of context. Eur J Immunol.

[42] Ma W, Ho DW, Sze KM, Tsui YM, Chan LK, Lee JM (2019). APOBEC3B promotes hepatocarcinogenesis and metastasis through novel deaminase-independent activity. Molecular carcinogenesis.

[43] Ho DW, Lam WM, Chan LK, Ng IO (2022). Investigation of Functional Synergism of CENPF and FOXM1 Identifies POLD1 as Downstream Target in Hepatocellular Carcinoma. Frontiers in medicine.

[44] Chiu YT, Husain A, Sze KM, Ho DW, Suarez EMS, Wang X (2023). Midline 1 interacting protein 1 promotes cancer metastasis through FOS-like 1-mediated matrix metalloproteinase 9 signaling in HCC. Hepatology.

[45] Husain A, Chiu YT, Sze KM, Ho DW, Tsui YM, Suarez EMS (2022). Ephrin-A3/EphA2 axis regulates cellular metabolic plasticity to enhance cancer stemness in hypoxic hepatocellular carcinoma. J Hepatol.

[46] Tsui YM, Sze KMF, Tung EKK, Ho DWH, Lee TKW, Ng IOL (2017). Dishevelled-3 phosphorylation is governed by HIPK2/PP1C alpha/ITCH axis and the non-phosphorylated form promotes cancer stemness via LGR5 in hepatocellular carcinoma. Oncotarget.

[47] Zhang VX, Sze KM, Chan LK, Ho DW, Tsui YM, Chiu YT (2021). Antioxidant supplements promote tumor formation and growth and confer drug resistance in hepatocellular carcinoma by reducing intracellular ROS and induction of TMBIM1. Cell & bioscience.

[48] Kam CS, Ho DW, Ming VS, Tian L, Sze KM, Zhang VX (2023). PFKFB4 Drives the Oncogenicity in TP53-Mutated Hepatocellular Carcinoma in a Phosphatase-Dependent Manner. Cell Mol Gastroenterol Hepatol.

[49] Chan LK, Ho DW, Kam CS, Chiu EY, Lo IL, Yau DT (2021). RSK2-inactivating mutations potentiate MAPK signaling and support cholesterol metabolism in hepatocellular carcinoma. J Hepatol.

[50] Stuart T, Butler A, Hoffman P, Hafemeister C, Papalexi E, Mauck WM 3rd (2019). Comprehensive Integration of Single-Cell Data. Cell.

[51] McGinnis CS, Murrow LM, Gartner ZJ (2019). DoubletFinder: Doublet Detection in Single-Cell RNA Sequencing Data Using Artificial Nearest Neighbors. Cell Syst.

[52] MacParland SA, Liu JC, Ma XZ, Innes BT, Bartczak AM, Gage BK (2018). Single cell RNA sequencing of human liver reveals distinct intrahepatic macrophage populations. Nat Commun.

[53] Ramilowski JA, Goldberg T, Harshbarger J, Kloppmann E, Lizio M, Satagopam VP (2015). A draft network of ligand-receptor-mediated multicellular signalling in human. Nat Commun.

