# Supplementary material for: Reciprocal interactions between malignant cells and macrophages enhance cancer stemness and M2 polarization in HBV-associated hepatocellular carcinoma
Source: Theranostics. 2024 Jan 1;14(2):892–910. doi: 10.7150/thno.87962 (PMC10758064; doi:10.7150/thno.87962)
Supplement: Supplementary file 1 — Supplementary figures and tables. [file thnov14p0892s1.pdf]

Figure S1. Cluster defining gene signatures in global landscape. (A) t-SNE plot of 43 clusters. Each point is a single cell colored by raw cluster assignment. **(B)** Heatmap showing the expression of top ten marker genes for each cluster. **(C)** t-SNE plot, color-coded for expression (gray to red) of immune cell marker gene (PTPRC/CD45). **(D)** t-SNE plot of all clusters. Each point is a single cell colored by patients. **(E)** Bar plot showing the fraction of immune cells belonging to each cluster for each patient. **(F)** t-SNE plot of all clusters for each patient. Each point is a single cell colored by raw cluster assignment.

Figure S2. Cluster characterization of cancer cells. (A) and **(B)** t-SNE plot of cancer cells. Each point is a single cell colored by raw cluster (A) and cluster assignment (B). **(C)** Heatmap showing the expression of top ten marker genes for each cluster. **(D)** Bar plot showing the fraction of cells belonging to each patient for each subpopulation. **(E)** The hierarchical heatmap showing large-scale CNVs of all chromosomes (columns) in cancer cell subsets.

Figure S3. Multi-color immunofluorescence staining of CD24, CD47 and ICAM1 in clinical specimen.

Figure S4. S100A11 knockdown efficiency and spheroid forming efficiency. S4RT-qPCR was performed for S100A11 in NTC and S100A11-knockdown cells for PLC/PRF/5 and CLC7 cell lines (mean ± SD).

Figure S5. CSCs-TAM crosstalk in HCC. (A) Heatmap showing the number of potential ligand-receptor pairs between TAMs subsets and cancer cells subsets (TAMs as receptors and cancer cells

as ligands). **(B)** Violin plots overview of M1/M2 canonical marker genes expression. **(C)** Box plot showing M1/M2 signature in subpopulations of macrophage. Boxplots represent the 25th and 75th percentiles, with midlines indicating the median values. The P-value was calculated using Wilcoxon's rank-sum test and shown at the top of each panel. * represents P-value < 0.05. **(D)** Pie chart showing relative proportion of macrophage subsets defined by marker genes. **(E)** Bar plot shows compositions of macrophage in each sample. Right Y axis shows the sum of the macrophage in each sample. **(F)** t-SNE plots highlight the distribution of $LGMN^{+}$/$SPP1^{+}$ TAMs and cancer cell cluster C6. Each dot corresponds to a single cell, colored according to cell type. **(G)** Venn diagrams of the ligand-receptor pairs between TAMs and cancer cells. **(H)** Bar plot depicts the top five enriched GO terms of ligand-receptor pairs. Y-axis represents the GO term, and the X-axis represents the enrichment significance (-log10 (*p*-value)). **(I)** Box plot showing M2 signature of each sample. Boxplots represent the 25th and 75th percentiles, with midlines indicating the median values.

Figure S6. Co-culture assays of macrophages and cancer cells, with siRNA knockdown of individual ligands on (A) cancer cells and (B) macrophages and examined the M1/M2 markers and liver cancer stem cell markers respectively, by qPCR.

Figure S7. qPCR revealed that knockdown of GAS6, ADAM9 and ANXA1 could result in downregulation of S100A11 in HCC cells.

Figure S8. In vivo S100A11 knockdown orthotopic liver injection mouse model and examination of tumor infiltrating macrophages.

Figure S9. Co-culture of macrophages with PLC/PRF/5 cells. (A), **(C)** and **(E)** For the co-culture assay, the components of macrophage M0/M1/M2 were measured by toluidine blue staining after co-cultured with PLC/PRF/5 cells for three days. **(B)**, **(D)** and **(F)** Bar plots shows migration level of M0/M1/M2 under different condition.

Figure S10. Authentication of cell lines used in this study. (A) Short tandem repeat (STR) DNA profiling of MHCC97L and CLC7 cells. **(B)** PCR confirmation of absence of murine contamination in PLC/PRF/5 and MHCC97L cells. Hepa1-6 is a mouse HCC cell line. Together with human (Hu) HCC samples, they were used as respective controls for testing human and mouse cell lines.

Supplementary Figures

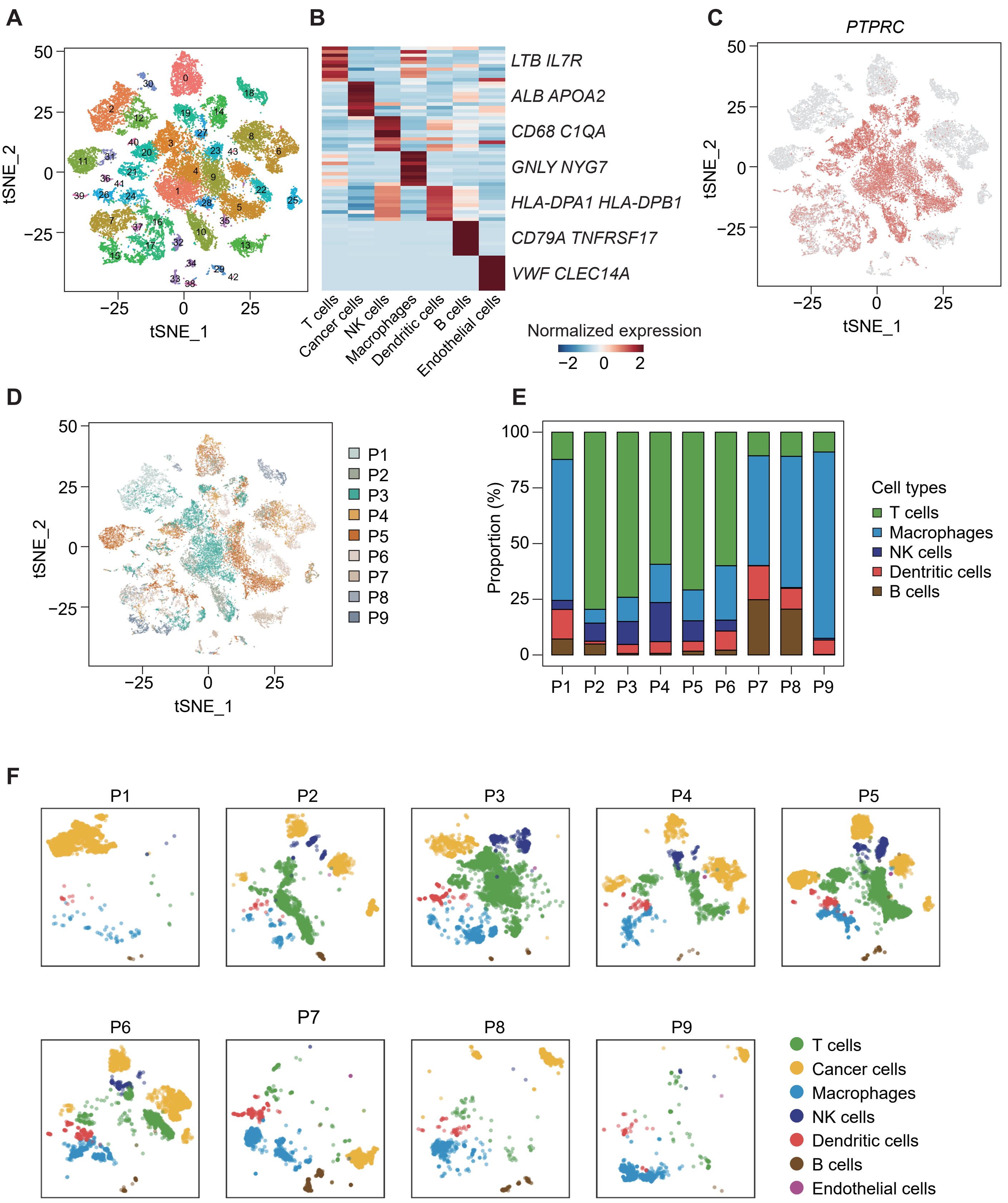

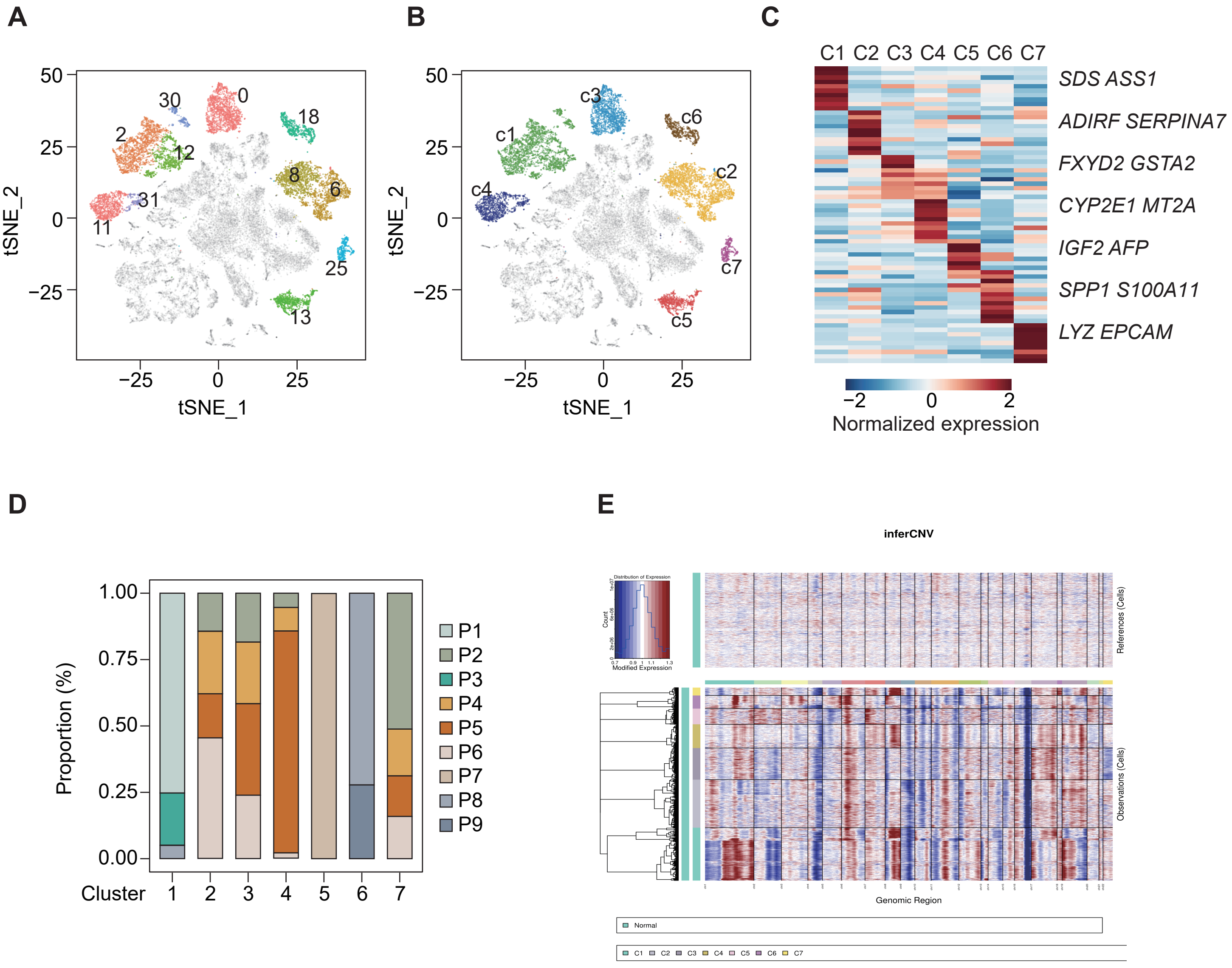
A
B
C
tSNE_2
tSNE_1
50
25
0
−25
30
0
2
18
12
8
6
31
11
25
13
c3
c6
c1
c2
c4
c7
c5
C1 C2 C3 C4 C5 C6 C7
SDS ASS1
ADIRF SERPINA7
FXYD2 GSTA2
CYP2E1 MT2A
IGF2 AFP
SPP1 S100A11
LYZ EPCAM
−2
0
2
Normalized expression
D
E
Proportion (%)
1.00
0.75
0.50
0.25
0.00
Cluster
1 2 3 4 5 6 7
P1
P2
P3
P4
P5
P6
P7
P8
P9
inferCNV
Distribution of Expression
Count
Modified Expression
References (Cells)
Observations (Cells)
Genomic Region
Normal
C1 C2 C3 C4 C5 C6 C7

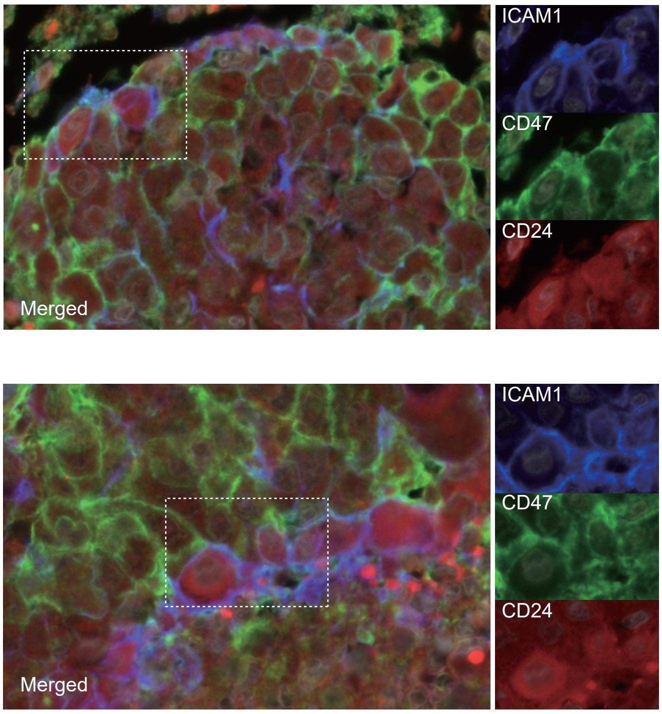
ICAM1
CD47
CD24
Merged
ICAM1
CD47
CD24
Merged

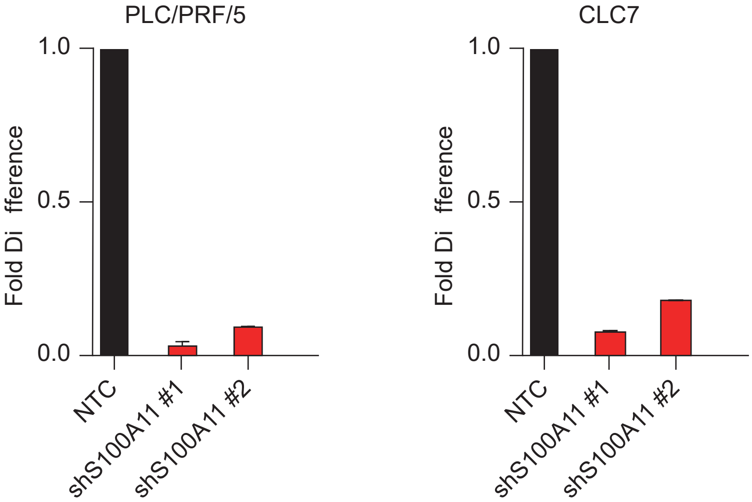

PLC/PRF/5
CLC7
Fold Di fference
1.0
0.5
0.0
NTC
shS100A11 #1
shS100A11 #2

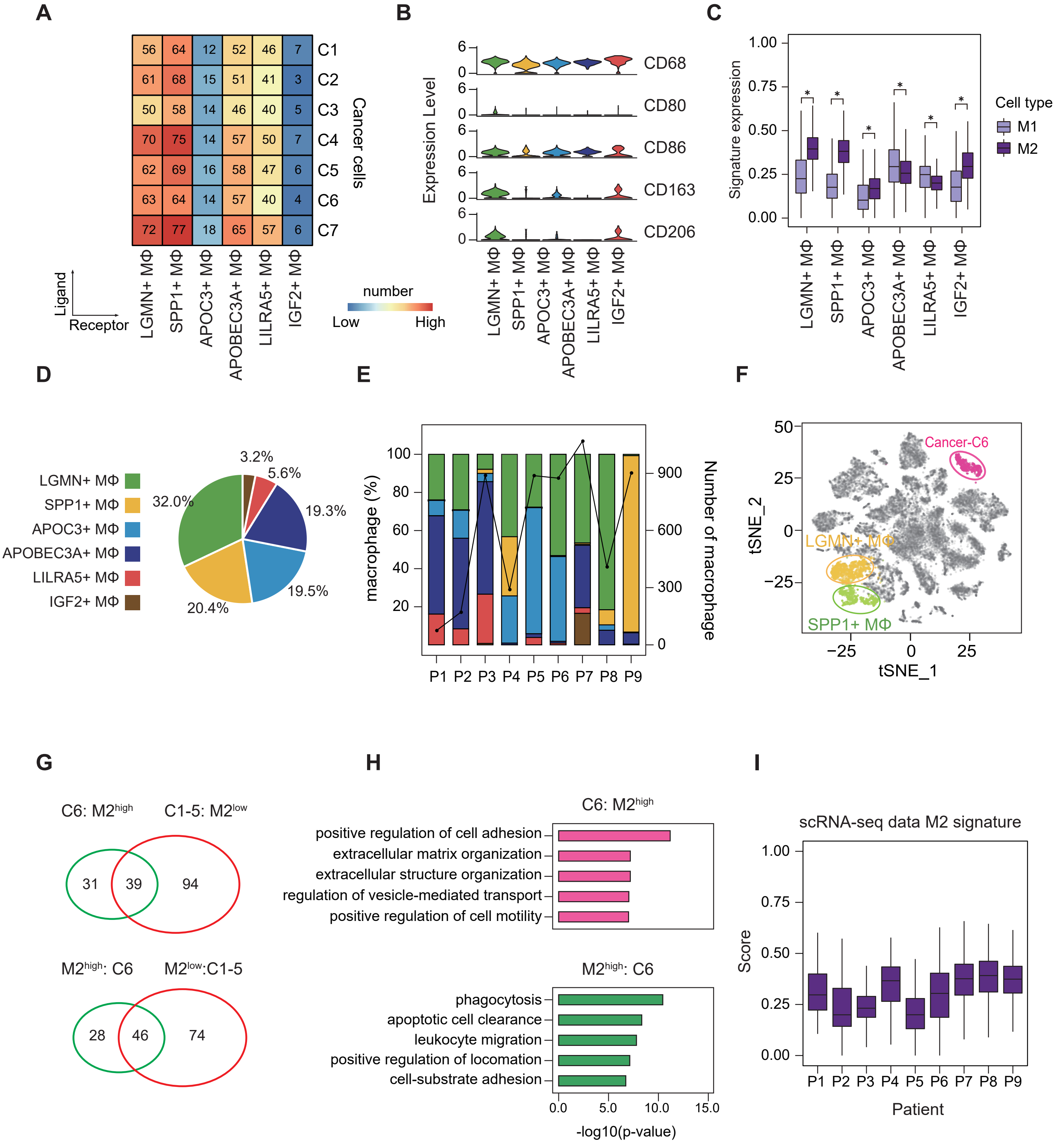
A
56 64 12 52 46 7 C1
61 68 15 51 41 3 C2
50 58 14 46 40 5 C3
70 75 14 57 50 7 C4
62 69 16 58 47 6 C5
63 64 14 57 40 4 C6
72 77 18 65 57 6 C7
Cancer cells
Ligand
Receptor
LGMN+ MΦ
SPP1+ MΦ
APOC3+ MΦ
APOBEC3A+ MΦ
LILRA5+ MΦ
IGF2+ MΦ
number
Low
High
B
Expression Level
CD68
CD80
CD86
CD163
CD206
C
Signature expression
1.00
0.75
0.50
0.25
0.00
Cell type
M1
M2
D
LGMN+ MΦ
SPP1+ MΦ
APOC3+ MΦ
APOBEC3A+ MΦ
LILRA5+ MΦ
IGF2+ MΦ
3.2%
5.6%
32.0%
19.3%
19.5%
20.4%
E
macrophage (%)
Number of macrophage
P1 P2 P3 P4 P5 P6 P7 P8 P9
F
Cancer-C6
LGMN+ MΦ
SPP1+ MΦ
tSNE_2
tSNE_1
G
C6: M2high
C1-5: M2low
31
39
94
M2high: C6
M2low:C1-5
28
46
74
H
C6: M2high
positive regulation of cell adhesion
extracellular matrix organization
extracellular structure organization
regulation of vesicle-mediated transport
positive regulation of cell motility
M2high: C6
phagocytosis
apoptotic cell clearance
leukocyte migration
positive regulation of locomotion
cell-substrate adhesion
-log10(p-value)
I
scRNA-seq data M2 signature
Score
Patient
P1 P2 P3 P4 P5 P6 P7 P8 P9

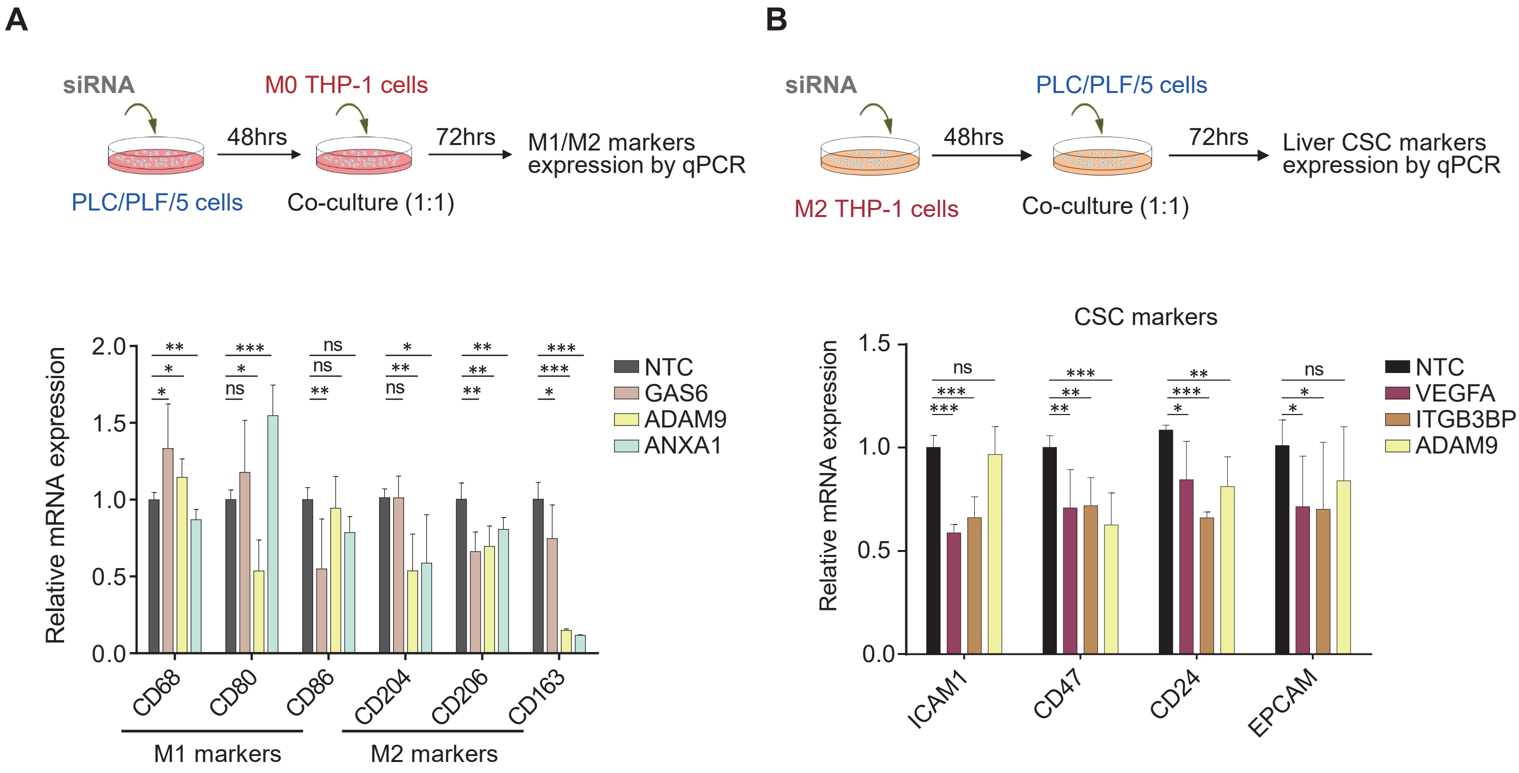

A
siRNA
M0 THP-1 cells
48hrs
72hrs
M1/M2 markers expression by qPCR
PLC/PLF/5 cells
Co-culture (1:1)
Relative mRNA expression
NTC
GAS6
ADAM9
ANXA1
CD68
CD80
CD86
CD204
CD206
CD163
M1 markers
M2 markers
B
siRNA
PLC/PLF/5 cells
48hrs
72hrs
Liver CSC markers expression by qPCR
M2 THP-1 cells
Co-culture (1:1)
CSC markers
Relative mRNA expression
NTC
VEGFA
ITGB3BP
ADAM9
ICAM1
CD47
CD24
EPCAM

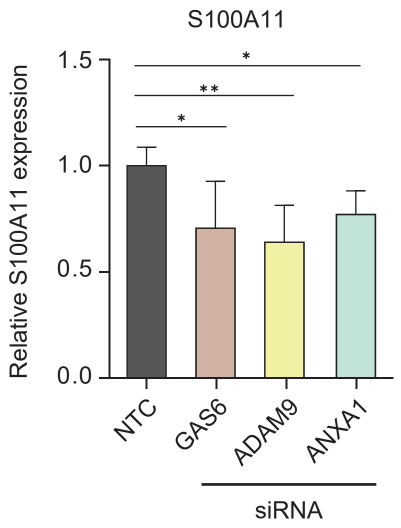

S100A11
Relative S100A11 expression
1.5
1.0
0.5
0.0
*
**
*
NTC
GAS6
ADAM9
ANXA1
siRNA

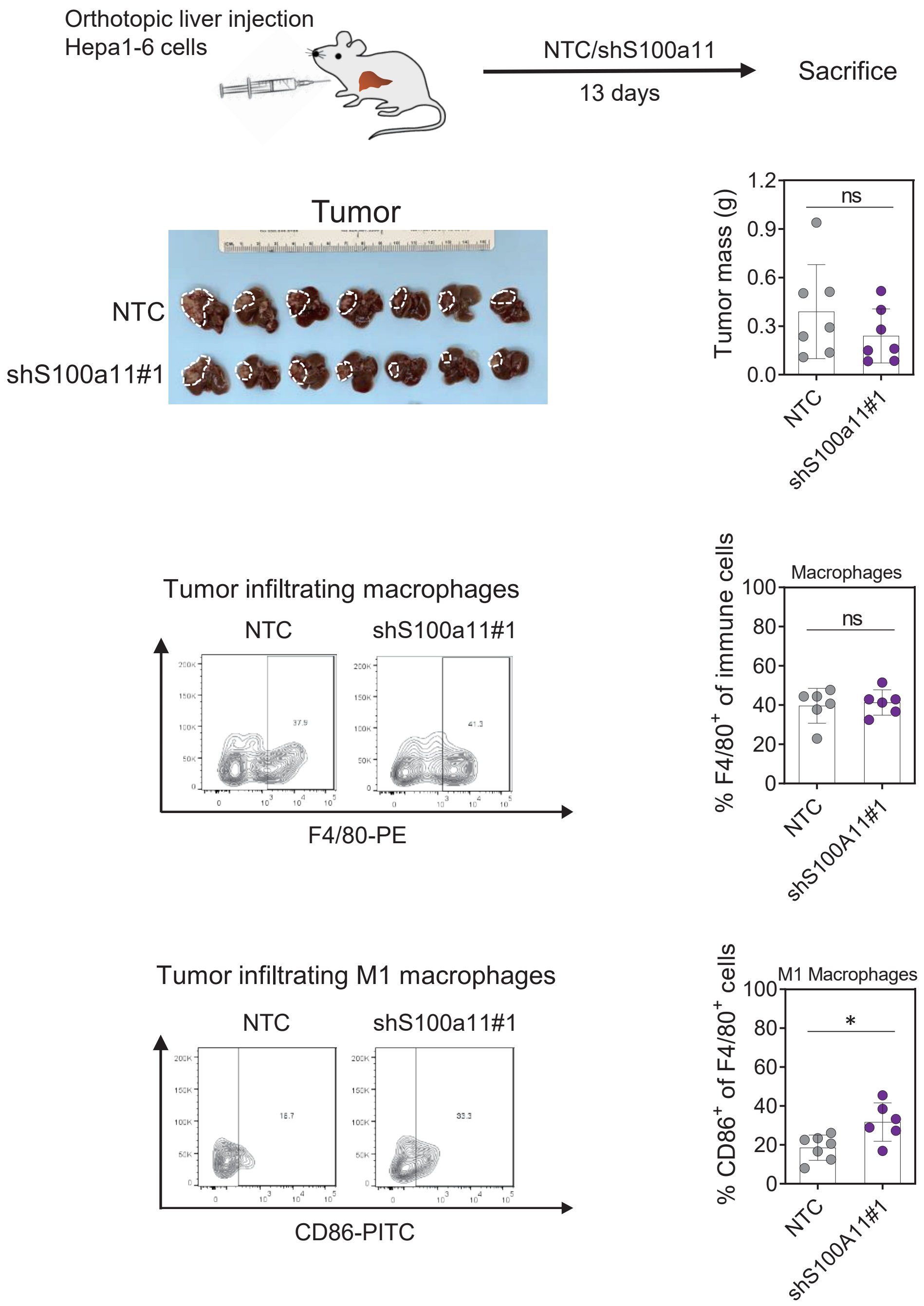

Orthotopic liver injection
Hepa1-6 cells
NTC/shS100a11
13 days
Sacrifice
Tumor
NTC
shS100a11#1
Tumor mass (g)
1.2
0.9
0.6
0.3
0.0
ns
NTC
shS100a11#1
Tumor infiltrating macrophages
NTC
shS100a11#1
F4/80-PE
Macrophages
% F4/80+ of immune cells
100
80
60
40
20
0
ns
NTC
shS100A11#1
Tumor infiltrating M1 macrophages
NTC
shS100a11#1
CD86-PITC
M1 Macrophages
% CD86+ of F4/80+ cells
100
80
60
40
20
0
*
NTC
shS100A11#1

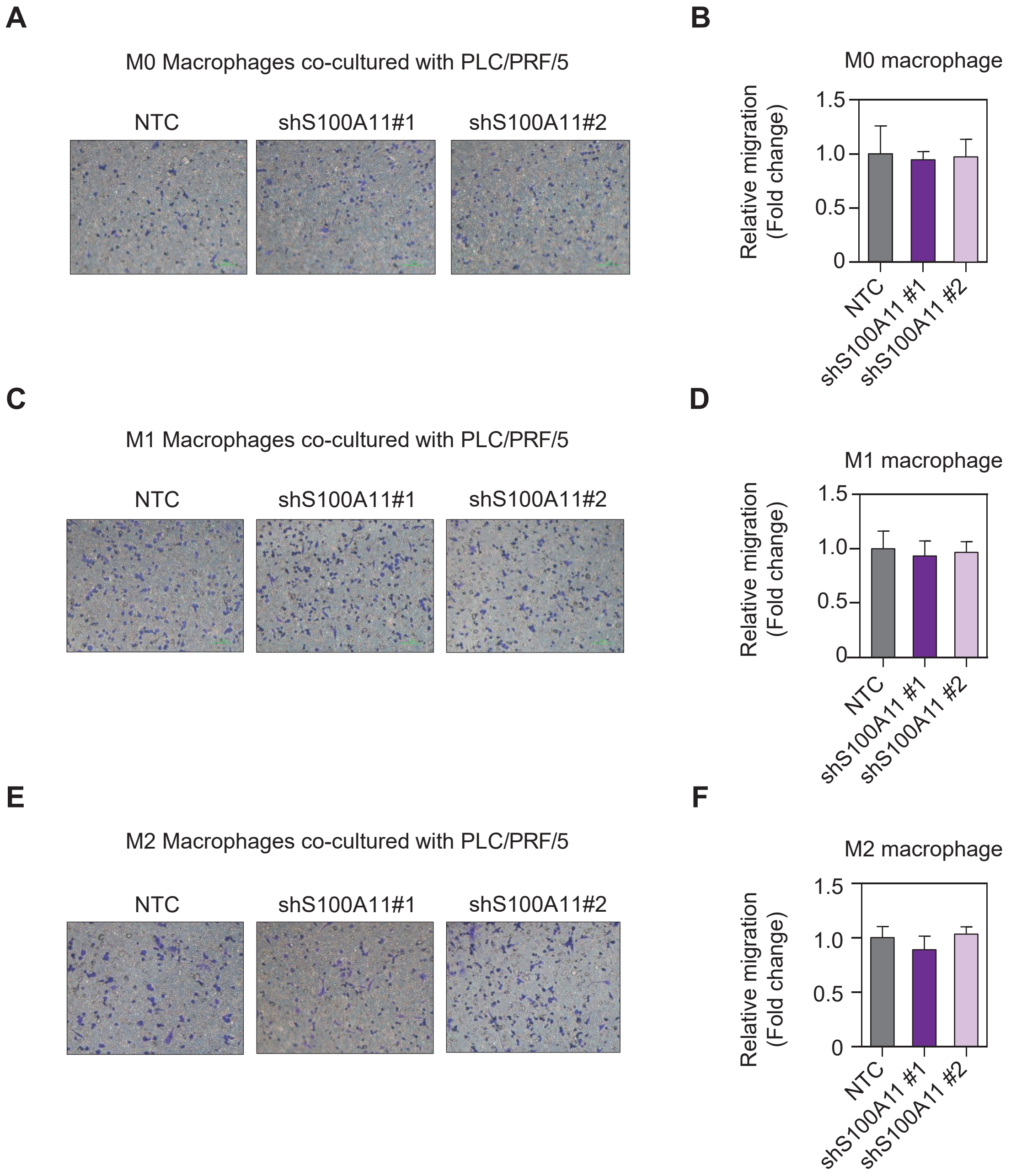

A
M0 Macrophages co-cultured with PLC/PRF/5
NTC
shS100A11#1
shS100A11#2
B
M0 macrophage
Relative migration (Fold change)
1.5
1.0
0.5
0
NTC
shS100A11 #1
shS100A11 #2
C
M1 Macrophages co-cultured with PLC/PRF/5
NTC
shS100A11#1
shS100A11#2
D
M1 macrophage
Relative migration (Fold change)
1.5
1.0
0.5
0
NTC
shS100A11 #1
shS100A11 #2
E
M2 Macrophages co-cultured with PLC/PRF/5
NTC
shS100A11#1
shS100A11#2
F
M2 macrophage
Relative migration (Fold change)
1.5
1.0
0.5
0
NTC
shS100A11 #1
shS100A11 #2

A

DNA Marker	MHCC97L[4]	MHCC97L (L-171218744P)
AMEL	X, Y	X, Y
CSF1PO	11, 13	11, 13
D13S317	8	8
D16S539	12	12
D5S818	12, 13	12, 13
D7S820	10	10
TH01	9	9
TPOX	8	8
vWA	14	14
D18S51	--	13, 22
D21S11	--	31.2
D3S1358	--	15, 16
D8S1179	--	12, 13
FGA	--	21, 24
Penta D	--	8, 9
Penta E	--	11, 17
Number of shared alleles		12
Total number of alleles in the reference profile		12
Percent match		100%

DNA Marker	CLC7[4]	CLC7(HCC) (L-230828748P)
AMEL	X	X
CSF1PO	13	13
D13S317	12	12
D16S539	9, 11	9, 11
D5S818	11, 13	11, 13
D7S820	10, 12	10, 12
TH01	7, 9	7
TPOX	8	8
vWA	14, 17	14, 17
D18S51	--	13, 14
D21S11	--	30, 33.2
D3S1358	--	15, 16
D8S1179	--	13
FGA	--	23
Penta D	--	9
Penta E	--	14,16
Number of shared alleles		13
Total number of alleles in the reference profile		14
Percent match		93%

B

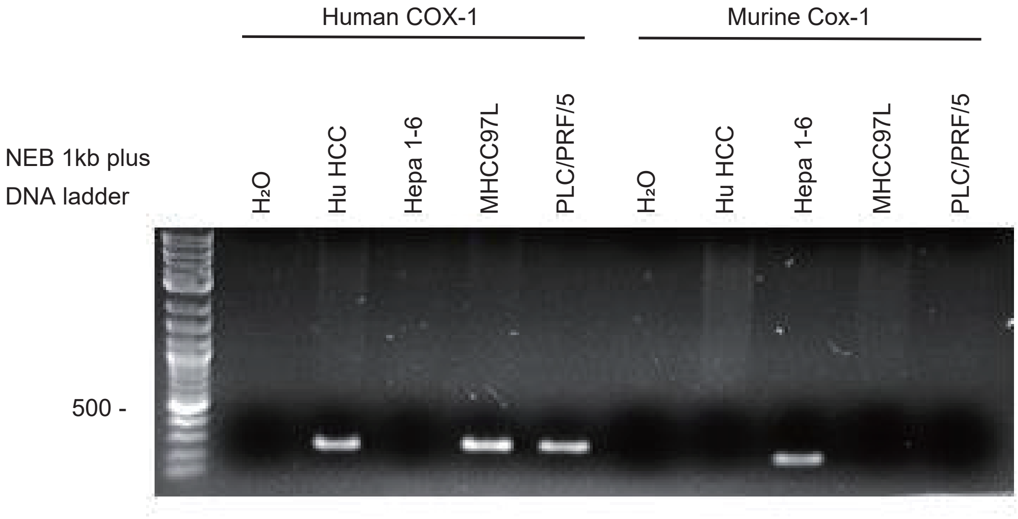

Supplementary Information

Supplementary Tables

Table S1. Demographic and clinical characteristics of the patients.

	P1	P2	P3	P4	P5	P6	P7	P8	P9
Gender	M	M	M	M	M	F	M	M	M
Age (years)	74	45	77	54	61	73	55	61	65
No. of tumor nodules	1	1	1	1	1	1	1	2	1
Tumor size (cm)	10	13	4.8	3.6	5	9	17	2	2.2
Cellular differentiation (Edmondson grading)	III	III-IV	II-III	III-IV	III-IV	III-IV	III	III-IV	III
Venous invasion	Present	Present	Absent	Present	Present	Present	Present	Present	Absent
Tumor microsatellite	Absent	Present	Absent	Present	Present	Present	Absent	Present	Absent
Liver invasion	Present	Present	Present	Absent	Present	Present	Present	Present	Present
Background liver	Chronic hepatitis	Chronic hepatitis	Cirrhosis	Cirrhosis	Cirrhosis	Chronic hepatitis	Chronic hepatitis	Cirrhosis	Chronic hepatitis
HBV status	Positive	Positive	Positive	Positive	Positive	Positive	Positive	Positive	Positive
HCV status	Negative	Negative	Negative	Negative	Negative	Negative	Negative	Negative	Negative
HBV DNA (IU/ml)	6.89×10^3	2.4×10^5	8.54×10^5	1700	1.9×10^6	<10	6.35×10^6	<10	3470
Hyperlipidemia	No	No	Unknown	No	No	Yes	No	No	No
Diabetes	Yes	No	No	No	No	Yes	No	No	No
Alcoholism	No	No	No	No	No	No	No	No	No

Table S2. Sequences for the oligos for cloning the shRNA constructs for establishing stable knockdown.

shRNAs	Oligos	
shS100A11 #1	Sense:	CCGGCAGCTAGATTTCTCAGAATTTCTCGAGAAATTCTGAGAAATCTAGCTGTTTTTG
	Antisense:	AATTCAAAAACAGCTAGATTTCTCAGAATTTCTCGAGAAATTCTGAGAAATCTAGCTG
shS100A11 #2	Sense:	CCGGGATTGCTGTCTTCCAGAAGTACTCGAGTACTTCTGGAAGACAGCAATCTTTTTG
	Antisense:	AATTCAAAAAGATTGCTGTCTTCCAGAAGTACTCGAGTACTTCTGGAAGACAGCAATC

Table S3. Primer sequences used for qRT-PCR analysis.

Target gene		Sequence
Human S100A11	Forward	GTGCATCGAGTCCCTGATTG
	Reverse	AGCTAGGCCACCAATCAGAT
Human ICAM1	Forward	AGCGGCTGACGTGTGCAGTAAT
	Reverse	TCTGAGACCTCTGGCTTCGTCA
Human CD24	Forward	GCTCCTACCCACGCAGATTT
	Reverse	GAGACCACGAAGAGACTGGC
Human CD47	Forward	CAATCACGTAAGGGTCTCATAGG
	Reverse	GATGGACTCCGATTTGGAGA
Human EPCAM	Forward	CCATGTGCTGGTGTGTGAAC
	Reverse	ACGCGTTGTGATCTCCTTCT
Human CD68	Forward	ATTCACCAGTTCTGCCCACC
	Reverse	GCTTCCCTGGACCTTGGTTT
Human CD80	Forward	TGCTGGCTGGTCTTTCTCAC
	Reverse	GTCCGGTTCTTGTACTCGGG
Human CD86	Forward	CCCCAGTGCACTATGGGAC
	Reverse	CAGGGTCCAACTGTCCGAAT
Human CD204	Forward	CGAAAGTTCGACTGGTCGGT
	Reverse	TGTCCCCCATTGCCGAATTT
Human CD206	Forward	CATCAGGGTGCAAGGAAGGT
	Reverse	TCCATCCGTCCAAAGGAACG
Human CD163	Forward	TCCTTGTGGGATTGTCCTGC
	Reverse	ATGGGAATTTTCTGCAAGCCG
Human HPRT	Forward	CTTTGCTGACCTGCTGGATT
	Reverse	CTGCATTGTTTTGCCAGTGT
